# Thyroid hormone sensitivity, insulin resistance and visceral fat area as determinants of prevalent coronary heart disease in euthyroid patients with type 2 diabetes

**DOI:** 10.3389/fendo.2026.1757587

**Published:** 2026-07-31

**Authors:** Xiaoxue Wang, Qiang Zhang, Hongli Liu, Yong Hu, Yimeng Wang, Yeju Wang, Kai Yang, Yijia Li

**Affiliations:** 1 Department of Endocrinology, Hanzhong Central Hospital, Hanzhong, Shaanxi, China; 2 Department of Radiation Oncology, Hanzhong Central Hospital, Hanzhong, Shaanxi, China

**Keywords:** coronary heart disease, insulin resistance, thyroid hormone sensitivity, type 2 diabetes, visceral fat area

## Abstract

**Background:**

Coronary heart disease (CHD) remains a leading cause of morbidity and mortality in patients with type 2 diabetes mellitus (T2DM). Whether thyroid hormone sensitivity indices add value for CHD risk stratification among euthyroid T2DM patients is unclear.

**Methods:**

In this retrospective study, we included 412 euthyroid adults with T2DM hospitalized at a tertiary hospital (112 with CHD and 300 without). We assessed thyroid hormone sensitivity indices (TT4RI, TFQI, TSHI and FT3/FT4 ratio), insulin resistance measures (HOMA-IR and METS-IR) and visceral fat area (VFA). Candidate predictors were screened by univariable logistic regression and collinearity diagnostics. A multivariable logistic regression model for prevalent CHD was developed using a stratified 70/30 train–validation split and evaluated with class-weighted stratified 5-fold cross-validation.

**Results:**

In univariable analyses, higher HOMA-IR, VFA, TT4RI, TSH, older age and hypertension were associated with CHD. Because TSH and TT4RI were highly collinear, TSH was excluded and TT4RI retained. In the final model, TT4RI, HOMA-IR, VFA and age remained independent predictors after accounting for hypertension. Discrimination was good (AUC 0.768 in the training set and 0.766 in the validation set; mean cross-validated AUROC 0.750 ± 0.035), with acceptable precision–recall performance (mean AUPRC 0.523 ± 0.074) and reasonable calibration.

**Conclusion:**

Impaired thyroid hormone sensitivity, increased insulin resistance and greater visceral fat accumulation are independently associated with prevalent CHD in euthyroid patients with T2DM. A simple model incorporating TT4RI, HOMA-IR and VFA may help refine CHD risk stratification in this population.

## Introduction

Type 2 diabetes mellitus (T2DM) is a metabolic disorder with high prevalence and significant mortality, representing a major public health challenge globally (1). In recent years, the incidence of T2DM has been increasing, especially in developing countries, where this trend is more pronounced (2). Coronary heart disease (CHD), a common complication of T2DM, is a leading cause of death in these patients. Studies have shown that individuals with T2DM are 2 to 4 times more likely to develop CHD than the general population is, with diabetes accelerating the progression of atherosclerosis (3). Early identification and assessment of prevalent CHD in T2DM patients are critical for improving patient prognosis and reducing complications (4).

Significant progress has been made in understanding the pathophysiology and risk factors for CHD in T2DM patients. Insulin resistance (IR) is a key pathological mechanism linking T2DM to CHD (5), leading to vascular endothelial dysfunction and accelerating atherosclerosis, thereby increasing prevalent CHD (6). Emerging metabolic indices, including the homeostasis model assessment of insulin resistance (HOMA-IR) and the metabolic score for insulin resistance (METS-IR), are widely used to assess IR and are strongly linked to prevalent CHD (7, 8). Additionally, the visceral fat area (VFA) is an important cardiovascular risk factor in T2DM patients, with larger visceral fat reserves correlating with higher CHD risk (9). Moreover, thyroid hormone alterations may contribute to prevalent CHD by influencing metabolism and fat distribution (10).

Thyroid hormone sensitivity, which reflects the body’s response to thyroid hormones, has gained increasing attention for its potential role in metabolic disorders, including T2DM and CHD (11–14). Variability in thyroid hormone sensitivity within the euthyroid range may reflect subtle alterations in central feedback regulation and/or peripheral thyroid hormone action. Such alterations can plausibly contribute to CHD risk in T2DM through several interconnected pathways, including dyslipidemia (hepatic cholesterol handling and lipoprotein metabolism), insulin resistance and adipose tissue dysfunction, endothelial dysfunction, vascular smooth muscle reactivity, and pro-inflammatory/pro-thrombotic states. Therefore, thyroid hormone sensitivity indices that integrate TSH and thyroid hormone levels (e.g., TT4RI, TFQI, and TSHI), together with a surrogate of peripheral conversion (FT3/FT4 ratio), may capture clinically meaningful heterogeneity beyond conventional thyroid function tests. Impaired thyroid hormone sensitivity has been linked to metabolic disturbances, such as insulin resistance and altered fat distribution—both of which are key factors in the development of CHD. This prompted us to focus on thyroid hormone sensitivity indices in our study. Indices such as the TT4RI (TT4 resistance index), Thyroid Feedback Quantile-based Index (TFQI), TSHI (thyroid sensitivity index) and FT3/FT4 ratio have been shown to correlate with various metabolic parameters, providing additional predictive value for assessing CHD risk in T2DM patients.

Despite these advancements, existing research on the relationships among IR, VFA, thyroid hormone sensitivity, and CHD risk in T2DM patients has several limitations. Most studies have focused on the impact of individual factors on CHD risk, with limited exploration of their combined effects or interactions. Furthermore, investigations into the role of thyroid hormone sensitivity in CHD risk, particularly among T2DM patients, are scarce. Therefore, there is a pressing need for further research into the multifactorial influences on prevalent CHD and the specific role of thyroid hormone sensitivity.

This study aimed to investigate the associations between thyroid hormone sensitivity, HOMA-IR, METS-IR, and VFA and the prevalent CHD in T2DM patients. By addressing gaps in the understanding of the combined effects of these factors, this study aimed to develop a predictive model for CHD in T2DM patients. Multivariate analyses will be conducted to identify key predictors of prevalent CHD, providing a scientific basis for the early identification of high-risk individuals. This study aimed to (1) examine the associations of thyroid hormone sensitivity indices, insulin resistance and visceral adiposity with prevalent CHD in euthyroid patients with T2DM, and (2) develop and internally validate a parsimonious prediction model for CHD risk stratification.

## Methods

### Study design and population

**Figure 1:**
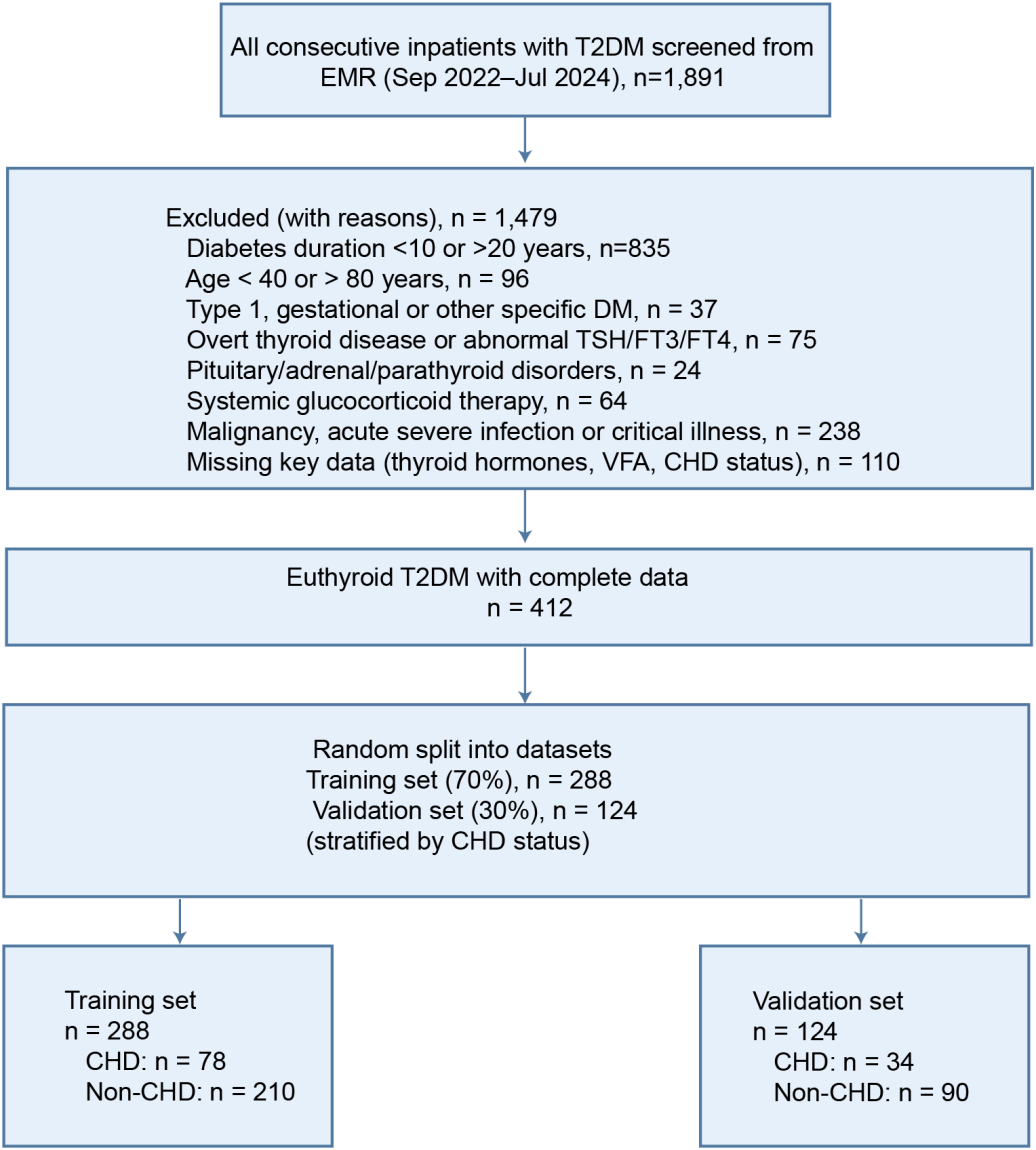
Study flow diagram. Flow of patient screening, exclusions and random stratified split into training (n=288) and validation (n=124) sets.

This retrospective, single-center study assessed the combined effects of thyroid hormone sensitivity, HOMA-IR, METS-IR, and VFA on prevalent CHD in patients with T2DM. A multivariate approach was used to analyze the clinical data and explore the relationships between these metabolic indices and CHD risk. This study included 412 patients with T2DM who were hospitalized in the Department of Endocrinology at Hanzhong Central Hospital between September 2022 and July 2024. To improve comparability and model stability, we restricted the cohort to patients aged 40–80 years because CHD events were rare in younger adults in our setting, which could lead to unstable estimates, and patients older than 80 years often had substantial comorbidity burden and heterogeneity that may introduce additional confounding and survival bias. To reduce heterogeneity in disease stage, we further restricted the cohort to patients with a diabetes duration of 10–20 years; this window was chosen to avoid too few CHD events in patients with very short diabetes duration and to limit confounding by multiple comorbidities in those with very long-standing T2DM. Because this was a retrospective study, the sample size was determined by the number of eligible patients with complete clinical, laboratory, and imaging data (including thyroid function tests and VFA measurements) available in the electronic medical record during the study period. Among these patients, 300 (70%) did not have CHD, whereas 112 (30%) had CHD. The flow of participant selection and random allocation to the training and validation sets is summarized in **Figure 1**.

### Patient identification and eligibility screening

We used a retrospective consecutive sampling strategy. Using the electronic medical record, we screened all consecutive inpatients with a diagnosis of type 2 diabetes admitted between September 2022 and July 2024 (n = 1,891). If a patient had multiple admissions during the study period, only the first eligible admission was retained to avoid duplicate observations. Overall, 1,479 patients were excluded and 412 were included in the final analytic cohort. To reduce heterogeneity in disease stage, we first restricted the screening pool to patients with a diabetes duration of 10–20 years, excluding those with duration <10 or >20 years (n = 835), leaving n = 1,056 patients for further eligibility screening. We then applied the remaining eligibility criteria (age 40–80 years and biochemical euthyroidism at admission) and prespecified exclusion criteria sequentially. Patients missing any key variable required for the primary analyses (thyroid hormones, fasting glucose/insulin, VFA, or definitive CHD ascertainment, including non-diagnostic/indeterminate coronary imaging reports) were excluded (complete-case analysis); no statistical imputation was performed. The numbers excluded at each step (with reasons) are summarized in **Figure 1**.

### Inclusion criteria

Age 40–80 years, with a diabetes duration of 10–20 years; a diagnosis of T2DM according to the 1999 World Health Organization (WHO) criteria; and a diagnosis of CHD after the onset of diabetes, defined as a history of coronary stent implantation, a previous myocardial infarction in a stable condition, or CHD confirmed by coronary angiography or coronary computed tomography angiography (CTA). Participants in the non-CHD group had no documented history of CHD/CAD, myocardial infarction, or coronary interventions, and were classified as non-CHD based on routine clinical evaluation and available non-invasive cardiac assessments during hospitalization (e.g., symptoms/history review, ECG, echocardiography, and cardiac imaging or stress testing when clinically indicated), reflecting real-world practice in our center. Coronary angiography or coronary CTA was performed only when clinically indicated.

### Exclusion criteria

Other types of diabetes, such as type 1 diabetes, gestational diabetes, or specific subtypes; acute diabetic complications or critical illness; thyroid disorders, use of thyroid-related medications, or history of thyroid surgery or radiation therapy; recent exposure to iodinated contrast agents (within 3 months prior to admission); use of glucocorticoid therapy (oral or intravenous) prior to or during hospitalization; disorders of the adrenal, pituitary, or parathyroid glands; significant cardiovascular disease, including acute myocardial infarction at presentation, acute or chronic heart failure (New York Heart Association functional class III–IV), hemodynamically significant arrhythmias, and other major cardiovascular or cerebrovascular conditions; severe infections, multiorgan failure, malignancies, or other major comorbidities.

This study adhered to the Declaration of Helsinki and was approved by the Ethics Committee of Hanzhong Central Hospital (Approval Number: 2024[131]).

### Admission indications and enrollment pathway

All participants were inpatients at the Department of Endocrinology. We retrospectively abstracted each patient’s primary admission indication from the electronic medical record (admission and discharge notes) and classified it into five pre-specified categories: cardiovascular evaluation/chest-pain work-up, diabetes complications management, mild infections, perioperative endocrine management (non-cardiac surgery) and outpatient poor glycemic control (no acute complications).

### Clinical data collection

#### General information

Patient data were obtained from the hospital’s electronic medical records. Baseline information included sex, age, medical history, and smoking and alcohol consumption habits. Smoking history was defined as continuous or cumulative smoking for six months or more at any point during the patient’s lifetime. Alcohol consumption was defined as drinking at least twice a week for more than one year. Height, weight, and blood pressure were measured and recorded by nursing staff at admission. Body mass index (BMI) was calculated and documented.

#### Medication use at presentation

Medication use at presentation was retrospectively abstracted from the patients’ electronic medical records. We collected data on the use of antidiabetic agents (insulin, metformin, sodium–glucose cotransporter-2 [SGLT2] inhibitors, glucagon-like peptide-1 receptor agonists [GLP-1RAs], dipeptidyl peptidase-4 [DPP-4] inhibitors, thiazolidinediones and sulfonylureas) and cardiovascular medications (statins, renin–angiotensin–aldosterone system inhibitors [RAASi, including ACE inhibitors and angiotensin receptor blockers], β-blockers, calcium channel blockers, diuretics and antiplatelet agents such as aspirin). Each medication class was coded as either used or not used at the time of hospital admission.

#### Laboratory measurements

Fasting venous blood samples were collected on the morning of the second hospital day after an 8–10 hour fast. The following biochemical parameters were measured: fasting blood glucose (FBG), glycated hemoglobin (HbA1c), fasting insulin (FINS), total cholesterol (TC), triglycerides (TG), high-density lipoprotein cholesterol (HDL-C), low-density lipoprotein cholesterol (LDL-C), thyroid-stimulating hormone (TSH), free triiodothyronine (FT3), and free thyroxine (FT4). Thyroid autoantibodies (TPOAb and TgAb) were not routinely assessed, because patients with known thyroid disease were excluded and all participants were biochemically euthyroid at admission. However, we cannot fully rule out euthyroid autoimmune thyroiditis. Fasting insulin was measured under routine treatment conditions; therefore, exogenous insulin therapy may have influenced HOMA-IR values in some patients. Biochemical euthyroidism was defined as serum TSH, FT3, and FT4 all within the institutional reference ranges (TSH 0.270–4.200 mIU/L; FT3 3.1–6.8 pmol/L; FT4 12–22 pmol/L).

#### Measurement of visceral fat area

The VFA was measured via bioelectrical impedance analysis (BIA). An experienced physician used a visceral fat analyzer (DUALSCAN HDS-2000, Omron) to measure the VFA.

#### Outcome ascertainment: CHD and non-CHD classification

CHD was ascertained from the electronic medical record and defined by (i) imaging-confirmed CHD on coronary CTA or invasive coronary angiography documented in a signed clinical report, (ii) a history of coronary stent implantation, or (iii) a prior myocardial infarction in a stable condition. Participants classified as non-CHD had no documented history of CHD/CAD, myocardial infarction, or coronary interventions.

In our center, coronary CTA and/or invasive angiography were performed under routine care when clinically indicated (e.g., chest tightness/discomfort, multiple cardiovascular risk factors, abnormal but non-diagnostic ECG/echocardiography findings, or specific clinical suspicion such as coronary artery origin anomalies). In the non-CHD group, coronary CTA was performed in 266/300 (88.7%) patients and invasive coronary angiography in 74/300 (24.7%), with some patients undergoing both tests. Non-diagnostic/indeterminate coronary imaging reports (e.g., non-evaluable segments due to image quality) were excluded because definitive CHD ascertainment was not possible.

#### Timing of index tests and coronary imaging

Fasting laboratory biomarkers were collected on the morning of hospital day 2 after an 8–10 h fast. VFA was assessed by bioelectrical impedance analysis during the index hospitalization, typically within the first 48 h after admission. For patients admitted for chest-pain evaluation, coronary CTA was typically scheduled after blood sampling on hospital day 2 so that thyroid and renal function assessment was completed before iodinated contrast exposure. When invasive coronary angiography was clinically indicated (e.g., high suspicion of CAD), it was performed during the index hospitalization according to routine care pathways. For patients with previously documented CHD (prior stent implantation, prior stable myocardial infarction, or prior coronary CTA/angiography confirming CHD), the reference standard was abstracted from existing medical records, whereas index tests (biomarkers and VFA) were measured at the current admission to characterise prevalent CHD status.

#### Coronary CTA and invasive coronary angiography: acquisition and interpretation

Coronary CTA was performed using standardized institutional protocols on ≥64-slice scanners, including a Siemens dual-source CT and a Canon 640-slice CT system. Because image quality improves with lower heart rates, heart-rate control (e.g., a β-blocker such as metoprolol) was administered when clinically appropriate and not contraindicated.

Invasive coronary angiography was performed under standard institutional workflows (radial or femoral access with routine multi-projection acquisition). Coronary stenosis severity was graded in routine clinical reports as mild (<50%), moderate (50%–69%), or severe (≥70%); stenosis ≥90% was reported as a critical finding according to institutional policy.

All coronary CTA and angiography studies were interpreted as part of routine care by board-certified cardiologists and/or radiologists with formal cardiovascular imaging training, using institutional reporting standards. Given the retrospective design, formal blinding of interpreters was not implemented; outcome ascertainment relied on the final signed clinical imaging reports archived in the electronic medical record.

#### Calculation of thyroid hormone sensitivity indices and metabolic indices

Thyroid hormone sensitivity indices (including TFQI, TSHI, TT4RI, and FT3/FT4 ratio) were calculated using established formulas (13, 15–18). The Thyroid Feedback Quantile-based Index (TFQI) ranges from −1 to 1. Negative values indicate preserved pituitary sensitivity to thyroid hormones, whereas positive values reflect impaired feedback regulation. For the Thyroid-Stimulating Hormone Index (TSHI) and Thyrotroph T4 Resistance Index (TT4RI), higher values correspond to reduced pituitary responsiveness to circulating thyroid hormones. The FT3/FT4 ratio was used as a surrogate marker of peripheral thyroid hormone conversion efficiency. A higher ratio suggests enhanced peripheral sensitivity to thyroid hormones. Metabolic indices, including the Homeostasis Model Assessment of Insulin Resistance (HOMA-IR) and the Metabolic Score for Insulin Resistance (METS-IR), were calculated using standard formulae as previously described.

### Statistical analysis

Statistical analyses were performed in Python 3.11 using the pandas, SciPy, statsmodels, seaborn, matplotlib and scikit-learn libraries. The nomogram was constructed separately in R version 4.4.3 using the rms package. Distributions of continuous variables were inspected visually using histograms and Q–Q plots to assess normality (**Supplementary Figure S1**). Continuous variables are summarized as mean ± standard deviation (SD) and were compared between patients with and without CHD using independent-samples t tests with unequal variances (Welch’s t tests). Categorical variables are expressed as counts and percentages and were compared using χ² tests, with Fisher’s exact tests applied when expected cell counts were small.

We first performed univariate logistic regression analyses in the full cohort to identify variables associated with CHD. Variables with p < 0.05 were considered candidates for multivariable modelling. Multicollinearity among candidate predictors was evaluated using the variance inflation factor (VIF) and correlation matrices. TSH and TT4RI exhibited pronounced collinearity (VIF ≈ 10), whereas the remaining predictors had acceptable VIF values. Therefore, TSH was excluded from further multivariable analyses and TT4RI was retained as the primary thyroid hormone sensitivity index. In the final predictor set (HOMA-IR, VFA, TT4RI, hypertension and age), all VIF values were <1.05, indicating negligible residual collinearity.

This was a retrospective study; therefore, no *a priori* sample size calculation was performed. To assess whether the available sample size was adequate for multivariable model development, we considered the number of CHD events relative to the number of parameters in the final model (events per variable, EPV). With 112 CHD events and 5 predictors retained in the final model (HOMA-IR, VFA, TT4RI, hypertension, and age), the EPV was 22.4, suggesting a favourable event-to-parameter ratio and a lower risk of overfitting. Model optimism was further mitigated by stratified train–validation splitting, class-weighted estimation, stratified 5-fold cross-validation, and calibration assessment (calibration plots and Brier score).

For model development, the dataset was randomly partitioned into a training set (70%) and an internal validation set (30%) using stratified sampling according to CHD status. CHD prevalence and key predictors (HOMA-IR, hypertension, age, VFA and TT4RI) were compared between the two sets to confirm the absence of material baseline differences. A multivariable logistic regression model including HOMA-IR, VFA, TT4RI, hypertension and age was fitted in the training set. Model coefficients were expressed as odds ratios (ORs) with 95% confidence intervals (CIs) and visualised using forest plots. The primary performance metric was discrimination quantified by the area under the receiver operating characteristic curve (AUROC), because it is threshold-independent and widely used for risk stratification models. As secondary metrics, we reported the area under the precision–recall curve (AUPRC) to reflect performance under class imbalance, and calibration using calibration plots and the Brier score. For descriptive, threshold-dependent summaries (sensitivity, specificity, accuracy and F1-score), we selected a single operating point by maximising Youden’s index in the training set and then applied the same threshold to the internal validation set to minimise optimistic bias.

To further characterise model performance and address class imbalance between CHD and non-CHD, we applied class-weighted estimation with stratified 5-fold cross-validation in the full dataset. All preprocessing steps were confined to the training folds to avoid information leakage. For each fold, we calculated AUROC and area under the precision–recall curve (AUPRC), as well as threshold-dependent metrics (accuracy, F1-score, sensitivity and specificity). Calibration was assessed using calibration plots and the Brier score. Fold-wise performance distributions are summarised graphically and in the **Supplementary Material**.

We performed a prespecified subgroup analysis among insulin-naïve patients (defined as no insulin use at admission). This analysis examined whether the associations and model performance were robust without potential distortion of HOMA-IR by exogenous insulin therapy. Continuous variables in the subgroup were standardized using the same z-score parameters derived from the full cohort to ensure comparability of effect estimates. The multivariable logistic regression model and performance metrics were evaluated using the same procedures as in the primary analysis.

Cardiometabolic medications may influence both thyroid hormone sensitivity and cardiovascular risk. Therefore, we performed sensitivity analyses with additional adjustment for medication use at admission. Specifically, binary indicators for the use of major glucose-lowering and cardiovascular drug classes (metformin, insulin, SGLT2 inhibitors, GLP-1RAs, DPP-4 inhibitors, thiazolidinediones, sulfonylureas, statins, aspirin, RAASi, β-blockers, calcium channel blockers and diuretics) were added to the model. These medication-adjusted models were fitted in the full cohort and used to assess the robustness of the associations of HOMA-IR, VFA, TT4RI, hypertension and age with CHD. Results are reported as ORs with 95% CIs in the **Supplementary Material**.

Our primary objective was risk stratification for prevalent CHD rather than causal inference. Therefore, we did not apply propensity score matching, inverse probability of treatment weighting or doubly robust estimators, as such distribution-altering approaches may distort baseline risk calibration and reduce external validity in prediction models. Instead, we preserved the observed outcome prevalence and used class-weighted cross-validation to mitigate imbalance, reporting both discrimination (AUROC, AUPRC) and calibration (Brier score).

## Results

### Baseline characteristics

This study enrolled 412 patients with type 2 diabetes mellitus (T2DM), comprising 112 with coronary heart disease (CHD) and 300 without. Key baseline characteristics of both groups are presented in **Table 1**. Compared with patients without CHD, those with CHD were older (62.89 ± 8.77 vs. 60.79 ± 8.25 years, p = 0.03) and had a higher prevalence of hypertension (60.7% vs. 41.7%, p = 0.001). The proportion of male patients was similar between groups (69.6% vs. 67.0%, p = 0.695), and diabetes duration did not differ significantly (14.04 ± 4.76 vs. 14.18 ± 2.65 years, p = 0.776).

**Table 1:** Baseline characteristics of patients with and without CHD.

Characteristics	Type	Overall	Diabetes without CHD	Diabetes with CHD	P
			(n=300)	(n=112)	
Demographic and clinical					
Age, years	continuous	61.36 ± 8.44	60.79 ± 8.25	62.89 ± 8.77	0.03
Gender (Male), n (%)	categorical	279 (67.7%)	201 (67.0%)	78 (69.6%)	0.695
Hypertension, yes (%)	categorical	193 (46.8%)	125 (41.7%)	68 (60.7%)	0.001
Diabetes duration, years	continuous	14.14 ± 3.35	14.18 ± 2.65	14.04 ± 4.76	0.776
Thyroid function					
TSH, mIU/L	continuous	2.24 ± 0.97	2.16 ± 0.92	2.47 ± 1.07	0.006
FT3, pmol/L	continuous	4.48 ± 0.60	4.48 ± 0.59	4.47 ± 0.63	0.82
FT4, pmol/L	continuous	16.37 ± 2.17	16.45 ± 2.04	16.16 ± 2.49	0.261
TT4RI	continuous	36.71 ± 16.62	35.49 ± 15.79	39.96 ± 18.34	0.024
Metabolic parameters					
HbA1c, %	continuous	8.53 ± 2.18	8.61 ± 2.18	8.30 ± 2.17	0.206
VFA, cm²	continuous	90.82 ± 38.49	87.58 ± 40.68	99.51 ± 30.38	0.001
HOMA_IR	continuous	2.04 ± 2.49	1.59 ± 2.01	3.25 ± 3.16	<0.001

Continuous variables are summarized as mean ± SD, and categorical variables as n (%). Between-group comparisons for continuous variables were performed using independent-samples t tests (Welch’s t test with unequal variances). Categorical variables were compared using the χ² test, with Fisher’s exact test applied when expected cell counts were small. All tests were two-sided. P values are reported to describe between-group differences and should not be interpreted as evidence of group comparability or causal associations because of the observational, non-randomized study design. The full list of baseline variables is provided in **Supplementary Table S1**.

With respect to thyroid-related indices, the CHD group had higher mean TSH concentrations (2.47 ± 1.07 vs. 2.16 ± 0.92 mIU/L, p = 0.006) and higher TT4RI values (39.96 ± 18.34 vs. 35.49 ± 15.79, p = 0.024), whereas FT3 and FT4 levels were comparable between groups. In terms of metabolic parameters, patients with CHD had greater visceral fat area (99.51 ± 30.38 vs. 87.58 ± 40.68 cm², p = 0.001) and higher HOMA-IR (3.25 ± 3.16 vs. 1.59 ± 2.01, p < 0.001), while HbA1c did not differ significantly. All p values correspond to between-group comparisons obtained using Welch’s t tests for continuous variables and χ² or Fisher’s exact tests for categorical variables, as detailed in the Methods. Comprehensive information on all baseline variables, including medication use, is provided in **Supplementary Table S1**.

### Admission indications

Reasons for admission fell into five pre-specified categories as summarised in **Supplementary Table S2**. Distributions are presented column-wise by CHD status (CHD n = 112; non-CHD n = 300). Cardiovascular evaluation/chest-pain work-up was more common in the CHD group (40.2% vs 5.0%), whereas outpatient poor glycaemic control and diabetes complications management were more frequent in the non-CHD group (30.0% vs 15.2% and 35.0% vs 19.6%, respectively). These variables were used to describe cohort context and were not included as predictors in multivariable models.

### Univariate analysis and multicollinearity diagnostics

Univariate logistic regression identified several factors significantly associated with CHD (**Table 2**): HOMA-IR (OR = 1.318, 95% CI: 1.184–1.466, p < 0.001), hypertension (OR = 2.164, 95% CI: 1.389–3.371, p < 0.001), TSH (OR = 1.395, 95% CI: 1.115–1.745, p = 0.004), VFA (OR = 1.008, 95% CI: 1.002–1.014, p = 0.006), TT4RI (OR = 1.016, 95% CI: 1.003–1.029, p = 0.016), and age (OR = 1.030, 95% CI: 1.004–1.057, p = 0.026). Other variables—including FT4, TSHI, FT3/FT4 ratio, TFQI, BMI, gender, smoking, FT3, drinking, and METS-IR—were not significantly associated with CHD (all p > 0.05). Although the FT3/FT4 ratio yielded an apparently high OR with a very wide confidence interval, the association was not statistically significant and the estimate was unstable; therefore, this variable was not considered a reliable predictor and was not carried forward into multivariable modelling. Multicollinearity was assessed using the variance inflation factor (VIF) and correlation matrix analyses (**Supplementary Figures S2A, SB**), which revealed strong collinearity between TSH and TT4RI (VIF ≈ 10). Consequently, TSH was excluded and TT4RI retained. After exclusion, all VIF values dropped below 1.05 (**Supplementary Figure S2C**), indicating negligible collinearity among the remaining variables. All predictors identified as significant in univariate analysis (except TSH) were subsequently included in the multivariable model. Univariable logistic regression was performed on the original (unstandardized) scale, whereas continuous predictors were standardized (z-scores) for multivariable model fitting.

**Table 2:** Univariate logistic regression analysis of factors associated with coronary heart disease in patients with type 2 diabetes mellitus.

Variable	OR (95% CI)	P-value
HOMA-IR	1.318 (1.184–1.466)	<0.001
Hypertension	2.164 (1.389–3.371)	<0.001
TSH	1.395 (1.115–1.745)	0.004
VFA	1.008 (1.002–1.014)	0.006
TT4RI	1.016 (1.003–1.029)	0.016
Age	1.030 (1.004–1.057)	0.026
FT4	0.932 (0.847–1.083)	0.218
TSHI	1.248 (0.853–1.824)	0.26
FT3/FT4 ratio	11.790 (0.122–1141.094)	0.29
TFQI	1.313 (0.769–2.244)	0.317
BMI	1.023 (0.947–1.206)	0.557
Gender (male)	1.132 (0.709–1.806)	0.609
Smoking	1.078 (0.658–1.764)	0.764
FT3	0.956 (0.667–1.375)	0.815
Drinking	1.042 (0.567–1.910)	0.896
METS-IR	1.001 (0.970–1.032)	0.943

### Model development and validation

The dataset was first randomly split into a training set (70%) and an internal validation set (30%). This split-sample approach was used for primary model development and evaluation. The prevalence of CHD and the distributions of key predictors, including HOMA-IR, hypertension, age, VFA and TT4RI, were similar between the two sets, as assessed by χ² tests for categorical variables and density plots for continuous variables (all p > 0.05; **Supplementary Figure S3**), suggesting that the random split did not introduce major imbalance in these variables.

**Figure 2:**
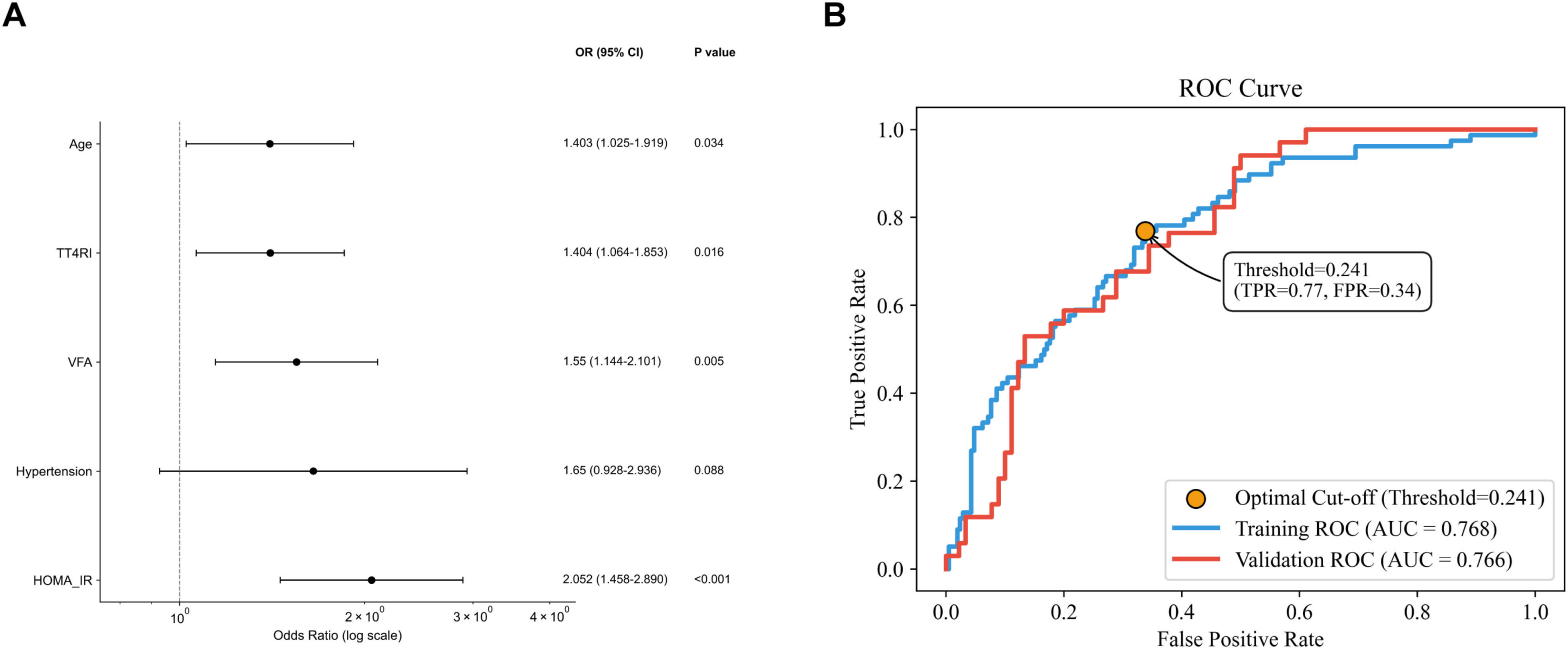
Multivariable predictors and discrimination of prevalent CHD in T2DM. **(A)** Forest plot of the multivariable logistic regression model in the training set. HOMA-IR, visceral fat area (VFA), TT4 resistance index (TT4RI) and age were statistically significant predictors of prevalent CHD, whereas hypertension showed a positive but non-significant association. Continuous predictors were standardised before modelling, so odds ratios correspond to a 1-standard-deviation increase. The x-axis is shown on a logarithmic scale. **(B)** Receiver operating characteristic (ROC) curves for the training and internal validation sets. The area under the curve (AUC) was 0.768 in the training set and 0.766 in the validation set. At the Youden-selected threshold of 0.241, sensitivity and specificity in the training set were 0.77 and 0.66, respectively.

Multivariable logistic regression including HOMA-IR, VFA, TT4RI, hypertension and age was then fitted in the training cohort to assess their independent associations with CHD in patients with T2DM. As shown in the forest plot (**Figure 2A**), higher HOMA-IR (OR = 2.052, 95% CI: 1.458–2.890, p < 0.001), VFA (OR = 1.550, 95% CI: 1.144–2.101, p = 0.005), TT4RI (OR = 1.404, 95% CI: 1.064–1.853, p = 0.016) and age (OR = 1.403, 95% CI: 1.025–1.919, p = 0.034) were independently associated with higher odds of prevalent CHD. Hypertension showed a positive but non-significant association (OR = 1.650, 95% CI: 0.928–2.936, p = 0.088) and was retained in the model because of its clinical relevance. All continuous predictors were standardised before modelling; thus, ORs for HOMA-IR, VFA, TT4RI and age correspond to a 1-SD increase in each variable.

**Figure 3:**
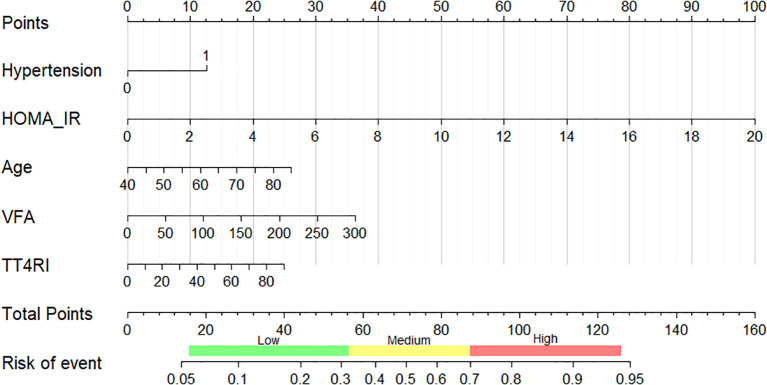
Nomogram for individualized estimation of prevalent CHD in T2DM. The nomogram incorporates hypertension, age, HOMA-IR, VFA, and TT4RI. For a given patient, align each predictor with the “Points” scale, sum the points to obtain “Total Points,” and project to the bottom scale to read the predicted probability of CHD. Higher total points indicate higher risk.

Within this split-sample framework, receiver operating characteristic (ROC) curve analysis demonstrated good discrimination, with similar areas under the curve (AUCs) in the training and validation sets (**Figure 2B**). At the optimal probability threshold determined in the training set, the model achieved a reasonable balance of sensitivity and specificity in both cohorts. Based on this multivariable model, a nomogram was developed to facilitate individualized estimation of the probability of prevalent CHD in patients with T2DM (**Figure 3**).

### Model performance

**Figure 4:**
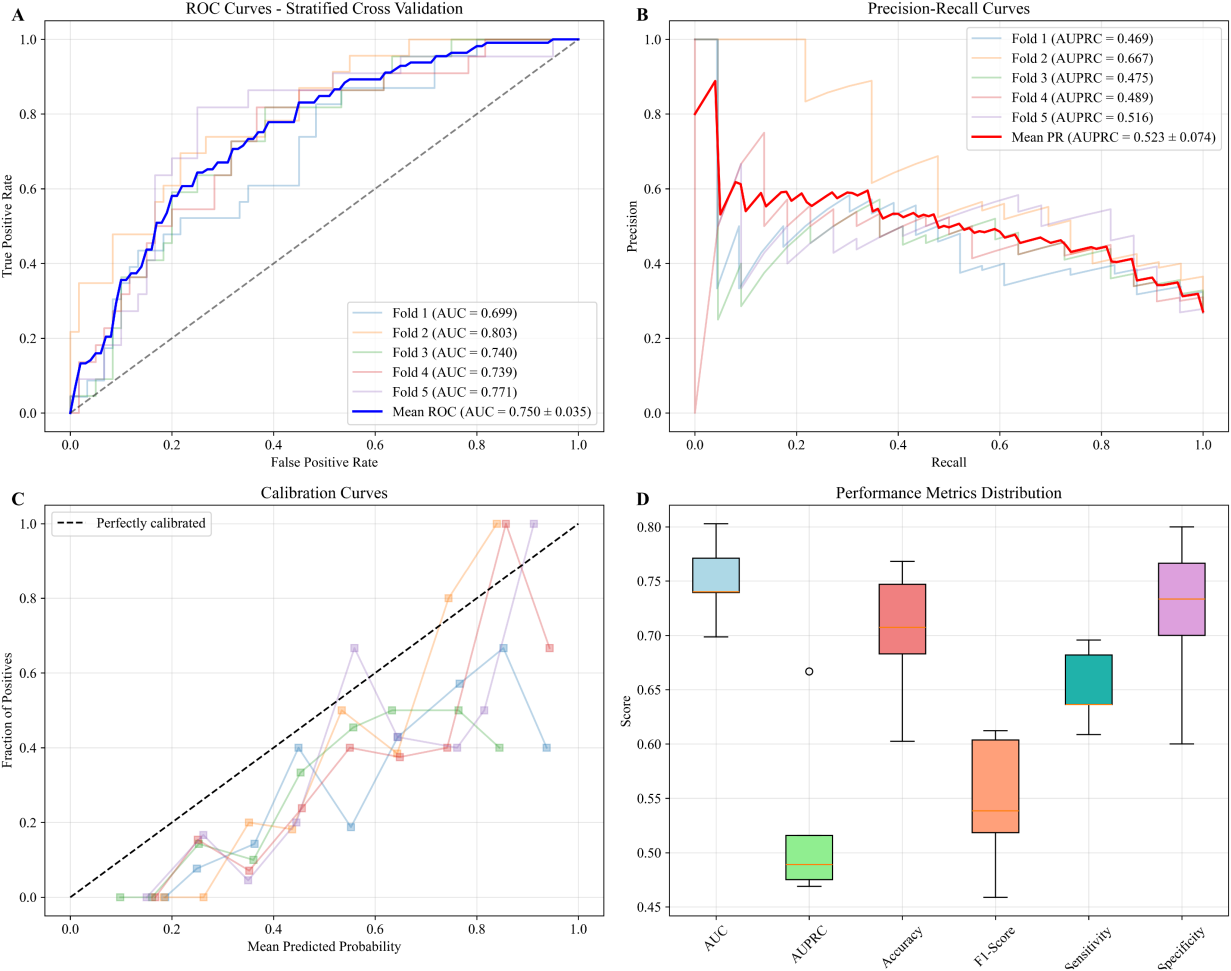
Cross-validated model performance (stratified 5-fold; class-weighted). **(A)** ROC curves (mean AUROC = 0.750 ± 0.035; AUROC, area under the ROC curve). **(B)** Precision–recall curves (mean AUPRC = 0.523 ± 0.074; AUPRC, area under the precision–recall curve). **(C)** Calibration curves showing agreement between predicted and observed risk. **(D)** Fold-wise distributions of key metrics (AUC, AUPRC, accuracy, F1-score, sensitivity, specificity).

Using class-weighted estimation with stratified 5-fold cross-validation, the model demonstrated stable performance despite class imbalance. The mean AUROC across folds was 0.750 ± 0.035 (**Figure 4A**), and the mean AUPRC was 0.523 ± 0.074 (**Figure 4B**). Calibration plots indicated acceptable agreement between predicted and observed risk (**Figure 4C**). The fold-wise distributions of key metrics—AUC, AUPRC, accuracy, F1-score, sensitivity, and specificity—further support model stability (**Figure 4D**).

### Sensitivity analyses adjusting for medications

Because cardiometabolic medications may influence thyroid hormone sensitivity, insulin resistance, and cardiovascular risk, we performed additional sensitivity analyses by extending the primary multivariable model to include major glucose-lowering and cardiovascular drug classes. Specifically, binary indicators for the use of metformin, insulin, SGLT2 inhibitors, GLP-1 receptor agonists, DPP-4 inhibitors, thiazolidinediones, sulfonylureas, statins, aspirin, renin–angiotensin–aldosterone system inhibitors, β-blockers, calcium channel blockers and diuretics at admission were added to the model.

In these medication-adjusted models, higher HOMA-IR and greater visceral fat area remained strongly associated with prevalent CHD, and hypertension continued to show an independent positive association with CHD. TT4RI also retained a positive but less precise association, with wider confidence intervals that crossed unity, whereas age was no longer significantly associated with CHD. Overall, the direction and magnitude of the associations for the main predictors were broadly similar to those of the primary model, indicating that our findings are not fully explained by differences in pharmacotherapy. Detailed coefficients from the medication-adjusted models are provided in **Supplementary Table S3**.

### Insulin-naïve subgroup analysis

In the insulin-naïve subgroup (n=203; CHD events=53), using the same z-score standardization parameters derived from the full cohort, HOMA-IR remained significantly associated with prevalent CHD (OR 1.907, 95% CI 1.257–2.891; P = 0.002), as did hypertension (OR 2.269, 95% CI 1.123–4.583; P = 0.022). TT4RI and visceral fat area showed directionally consistent but non-significant associations, while age demonstrated a borderline association (P = 0.082). Model discrimination was preserved, with an apparent AUROC of 0.745 and a mean 5-fold cross-validated AUROC of 0.733 ± 0.069. Precision–recall performance was comparable (apparent AUPRC 0.514; mean cross-validated AUPRC 0.511 ± 0.104) (**Supplementary Figure S4**).

## Discussion

In this retrospective hospital-based study of euthyroid inpatients with T2DM, we found that impaired thyroid hormone sensitivity, increased insulin resistance and greater visceral fat accumulation were all associated with prevalent CHD. In multivariable models, higher HOMA-IR, larger VFA, higher TT4RI and older age remained independent predictors of CHD after accounting for hypertension. Hypertension showed a positive but borderline non-significant association and was retained in the model because of its clinical relevance. The direction and magnitude of these associations were generally preserved in sensitivity analyses additionally adjusting for major glucose-lowering and cardiovascular medications, suggesting that our findings are not fully explained by differences in pharmacotherapy. Taken together, our results support a multifactorial framework in which thyroid hormone sensitivity, visceral adiposity, insulin resistance and age-related vascular changes may jointly contribute to the burden of CHD in T2DM.

Age and hypertension are well-established risk factors for CHD. In our study, patients with T2DM and CHD were significantly older than those without CHD, consistent with evidence that vascular stiffening and age-related metabolic disturbances promote atherosclerosis and coronary events (19, 20). A history of hypertension was also more frequent among patients with CHD, in line with data showing that elevated blood pressure increases prevalent CHD by increasing cardiac workload, accelerating endothelial dysfunction and fostering plaque formation (21, 22). In the final multivariable model, age remained independently associated with CHD, whereas hypertension showed a positive but non-significant association. Both variables were therefore retained in the prediction model and nomogram because of their strong biological plausibility and established prognostic importance.

In univariate analyses, higher TSH levels within the reference range were associated with CHD, even though all participants were biochemically euthyroid. Mechanistic links between TSH and atherosclerosis—mediated through adverse lipid remodelling, oxidative stress and endothelial dysfunction—have been most clearly demonstrated in subclinical hypothyroidism, particularly when TSH exceeds 10 mIU/L, and may not directly generalise to euthyroid states (23–27). Nevertheless, several studies have reported that higher “high-normal” TSH is associated with a more atherogenic lipid profile and with cardiovascular events and mortality in diabetes and general-population cohorts (28–30). These reports are directionally consistent with our unadjusted findings. However, TSH was strongly collinear with TT4RI in our data (VIF ≈ 10) and was therefore excluded from multivariable analyses. We deliberately focused on thyroid hormone sensitivity indices as our construct of interest, treating TT4RI as the primary marker of central (pituitary) thyroid hormone responsiveness. Thyroid hormone sensitivity indices—including TFQI, TSHI, TT4RI and the FT3/FT4 ratio—capture subtle impairments in feedback regulation and peripheral conversion that are not apparent from conventional thyroid function tests alone (15, 27). In our cohort, higher TT4RI was independently associated with CHD, reinforcing emerging evidence that reduced sensitivity to thyroid hormones, even within the euthyroid range, is linked to cardiometabolic disease and adverse cardiovascular outcomes (31, 32). Mechanistically, diminished sensitivity may impair lipid metabolism and promote visceral adiposity, thereby aggravating insulin resistance and triggering pro-inflammatory cytokine release (e.g. TNF-α, IL-6), which together foster atherosclerosis (33–36). Future prospective studies should determine whether TSH adds incremental prognostic value beyond thyroid hormone sensitivity indices in euthyroid patients with T2DM.

The VFA emerged as another key factor associated with CHD. Visceral adiposity is a core component of metabolic syndrome and is closely linked to insulin resistance, dyslipidaemia and chronic low-grade inflammation—all major determinants of atherosclerotic disease (37, 38). Visceral fat depots secrete adipokines and inflammatory mediators, promote oxidative stress and impair endothelial function, which in turn accelerate plaque formation and vascular remodelling (39, 40). Our findings add to previous work by highlighting that, among euthyroid patients with long-standing T2DM, quantitative assessment of VFA—rather than BMI alone—may provide important additional information for cardiovascular risk stratification.

HOMA-IR was identified as a significant predictor of CHD in this study, underscoring the central role of insulin resistance in diabetes-related macrovascular disease. Insulin resistance contributes to CHD through multiple converging mechanisms, including disturbances in lipid metabolism, increased inflammation, impaired endothelial function and enhanced thrombogenicity (41–45). These pathophysiological changes accelerate atherosclerosis and destabilise existing plaques. In contrast, METS-IR did not emerge as a significant predictor of CHD in our cohort, despite being a validated surrogate of insulin resistance in other settings (17, 38). A plausible explanation is that all participants had received long-term lifestyle and pharmacological interventions, and patients with CHD were more likely to receive standard secondary-prevention therapies such as statins and antihypertensives. As a result, between-group differences in glycaemic control, lipid profiles and BMI at admission were attenuated, potentially blunting the discriminatory capacity of METS-IR, which depends heavily on these components. In this context, HOMA-IR—derived from fasting glucose and insulin—may better capture residual insulin resistance that is not fully corrected by treatment.

The final multivariable model integrating HOMA-IR, VFA, TT4RI and age (with hypertension retained for clinical interpretability) demonstrated moderate discrimination, with AUROC values of 0.768 and 0.766 in the training and validation sets, respectively, and maintained acceptable calibration in cross-validated analyses. The nomogram derived from this model may facilitate individualized CHD probability estimation in T2DM, particularly in inpatient or tertiary-care settings where detailed metabolic assessment is routinely available. Importantly, sensitivity analyses adjusting for cardiometabolic medications yielded effect estimates that were broadly consistent with those of the primary model, indicating that the associations of HOMA-IR, VFA and TT4RI with CHD are robust to differences in pharmacotherapy.

From a clinical perspective, TT4RI (a thyroid hormone sensitivity index), together with HOMA-IR and visceral adiposity (VFA), may help refine CHD risk stratification among euthyroid patients with T2DM beyond conventional risk factors. Because TT4RI can be derived from routine thyroid function tests, it is potentially scalable in real-world practice. If confirmed in prospective cohorts, incorporating thyroid hormone sensitivity metrics alongside established predictors may support more individualized cardiovascular prevention and closer monitoring in selected higher-risk individuals. However, given the observational and cross-sectional nature of prevalent CHD in this study, these indices should be interpreted as risk markers rather than treatment targets.

Future studies should validate these findings in independent multi-center cohorts and, ideally, prospective longitudinal designs to establish temporality and evaluate prediction of incident CHD events. Mechanistic work integrating inflammatory biomarkers, insulin resistance phenotypes and imaging-based measures of atherosclerosis may further clarify pathways linking thyroid hormone sensitivity to CHD in T2DM. Repeated measurements may also determine whether dynamic changes in thyroid hormone sensitivity improve risk prediction beyond single time-point assessment.

Established cardiovascular risk scores (e.g., ASCVD- or Framingham-type tools) primarily rely on traditional factors such as age, sex, blood pressure, smoking status, and lipid profiles, and are mainly designed to predict future cardiovascular events in the general population. In contrast, our model was developed specifically in euthyroid patients with type 2 diabetes and incorporates thyroid hormone sensitivity (TT4RI), insulin resistance (HOMA-IR), and visceral adiposity (VFA), which may capture metabolic heterogeneity not fully reflected by conventional predictors. Therefore, our model is not intended to replace existing risk scores; rather, it may serve as a complementary tool for risk stratification in inpatient or tertiary-care settings where these measurements are routinely available. Prospective studies directly comparing the incremental value of this model over standard risk scores are warranted.

This study has several strengths. We focused on a carefully characterised inpatient cohort of euthyroid patients with T2DM with detailed information on thyroid hormone sensitivity indices, insulin resistance markers, VFA and medication use. We applied rigorous statistical procedures, including multicollinearity diagnostics, class-weighted stratified cross-validation, and complementary discrimination and calibration metrics, to develop and internally validate a parsimonious prediction model.

Nevertheless, several limitations must be acknowledged. First, the retrospective, single-centre design may introduce selection and information bias and limits generalisability beyond similar tertiary-care settings. Second, because the primary analyses were based on complete cases, selection bias cannot be excluded, as included patients had complete thyroid function tests, VFA measurement and CHD ascertainment required for modelling. Third, the timing of coronary imaging relative to index tests was not fully standardised across patients; although measurements were obtained within routine inpatient workflows, heterogeneity in time intervals may exist. Fourth, CHD was defined as prevalent disease at baseline rather than incident events, precluding causal inference and temporality assessment. Fifth, although coronary CTA/angiography was performed under clinical indications rather than a study-mandated protocol, some subclinical or asymptomatic CHD may still have been missed, leading to potential outcome misclassification. Such misclassification would be expected to attenuate observed associations and reduce discrimination (bias toward the null), suggesting that our estimates may be conservative. Prospective studies with standardised CHD adjudication are warranted. Sixth, medication exposure was captured at admission only and does not reflect cumulative dose or long-term adherence; residual confounding by treatment intensity or prior therapy cannot be excluded despite our sensitivity analyses. Seventh, thyroid autoantibodies and other markers of thyroid autoimmunity were not assessed, although patients with known thyroid disease were excluded. Eighth, HOMA-IR was calculated in a hospitalised cohort in which many patients were receiving exogenous insulin, which may attenuate its ability to fully capture endogenous insulin resistance in some individuals. Finally, external validation in independent cohorts and prospective studies with longer follow-up are needed to confirm the stability and transportability of our prediction model.

## Conclusion

In euthyroid patients with T2DM, impaired thyroid hormone sensitivity (higher TT4RI), increased insulin resistance and greater visceral fat accumulation were independently associated with prevalent CHD after accounting for age and hypertension; these associations were generally robust in sensitivity analyses additionally adjusting for cardiometabolic medications. A parsimonious model incorporating TT4RI, HOMA-IR and VFA demonstrated moderate discrimination and acceptable calibration, supporting their potential utility for CHD risk stratification. Prospective multi-center validation is warranted.

## Data Availability

The raw data supporting the conclusions of this article will be made available by the authors, without undue reservation.

## Supplementary material

Supplementary Table 1 Baseline characteristics and medication use of euthyroid patients with type 2 diabetes according to coronary heart disease status. Data are presented as mean ± SD for continuous variables; for variables with skewed distributions, medians and interquartile ranges (IQRs) are additionally reported. Categorical variables are summarised as n (%). Between-group comparisons for continuous variables were performed using independent-samples t tests with unequal variances (Welch’s t tests). Categorical variables were compared using χ² tests, with Fisher’s exact tests applied when expected cell counts were small. All tests were two-sided. BMI, body mass index; FBG, fasting blood glucose; HDL-C, high-density lipoprotein cholesterol; LDL-C, low-density lipoprotein cholesterol; TC, total cholesterol; TG, triglycerides; VFA, visceral fat area; HOMA-IR, homeostasis model assessment of insulin resistance; TyG, triglyceride–glucose index; METS-IR, metabolic score for insulin resistance; TFQI, thyroid feedback quantile-based index; PTFQI, parametric TFQI; TSHI, TSH index; TT4RI, thyrotroph T4 resistance index; RAASi, renin–angiotensin–aldosterone system inhibitor; DPP-4i, dipeptidyl peptidase-4 inhibitor; GLP-1RA, glucagon-like peptide-1 receptor agonist; SGLT2i, sodium–glucose cotransporter-2 inhibitor; TZD, thiazolidinedione.

Supplementary Table 2 Distribution of admission reasons by CHD status. Admission reasons were retrospectively abstracted and classified into five predefined categories. Percentages are column-wise (CHD: n=112; non-CHD: n=300; overall: n=412). One primary reason per patient. Variables were used for cohort description only and were not entered into multivariable modelling.

Supplementary Table 3 Sensitivity analyses of the association between key predictors and prevalent CHD before and after adjustment for medication use. * Primary multivariable logistic regression model including HOMA-IR, VFA, TT4RI, hypertension and age. † Medication-adjusted model additionally including binary indicators for the use of metformin, insulin, SGLT2 inhibitors, GLP-1 receptor agonists, DPP-4 inhibitors, thiazolidinediones, sulfonylureas, statins, aspirin, renin–angiotensin–aldosterone system inhibitors, β-blockers, calcium channel blockers and diuretics at admission.OR, odds ratio; CI, confidence interval; VFA, visceral fat area; TT4RI, thyrotroph T4 resistance index. Continuous predictors were standardised before modelling; ORs therefore correspond to a change in the standardised scale rather than the raw clinical units.
