# Supplementary material for: Thyroid hormone sensitivity, insulin resistance and visceral fat area as determinants of prevalent coronary heart disease in euthyroid patients with type 2 diabetes
Source: Front Endocrinol (Lausanne). 2026 Jul 31;17:1757587. doi: 10.3389/fendo.2026.1757587 (PMC13472804; doi:10.3389/fendo.2026.1757587)
Supplement: Supplementary file 1 [file DataSheet1.pdf]

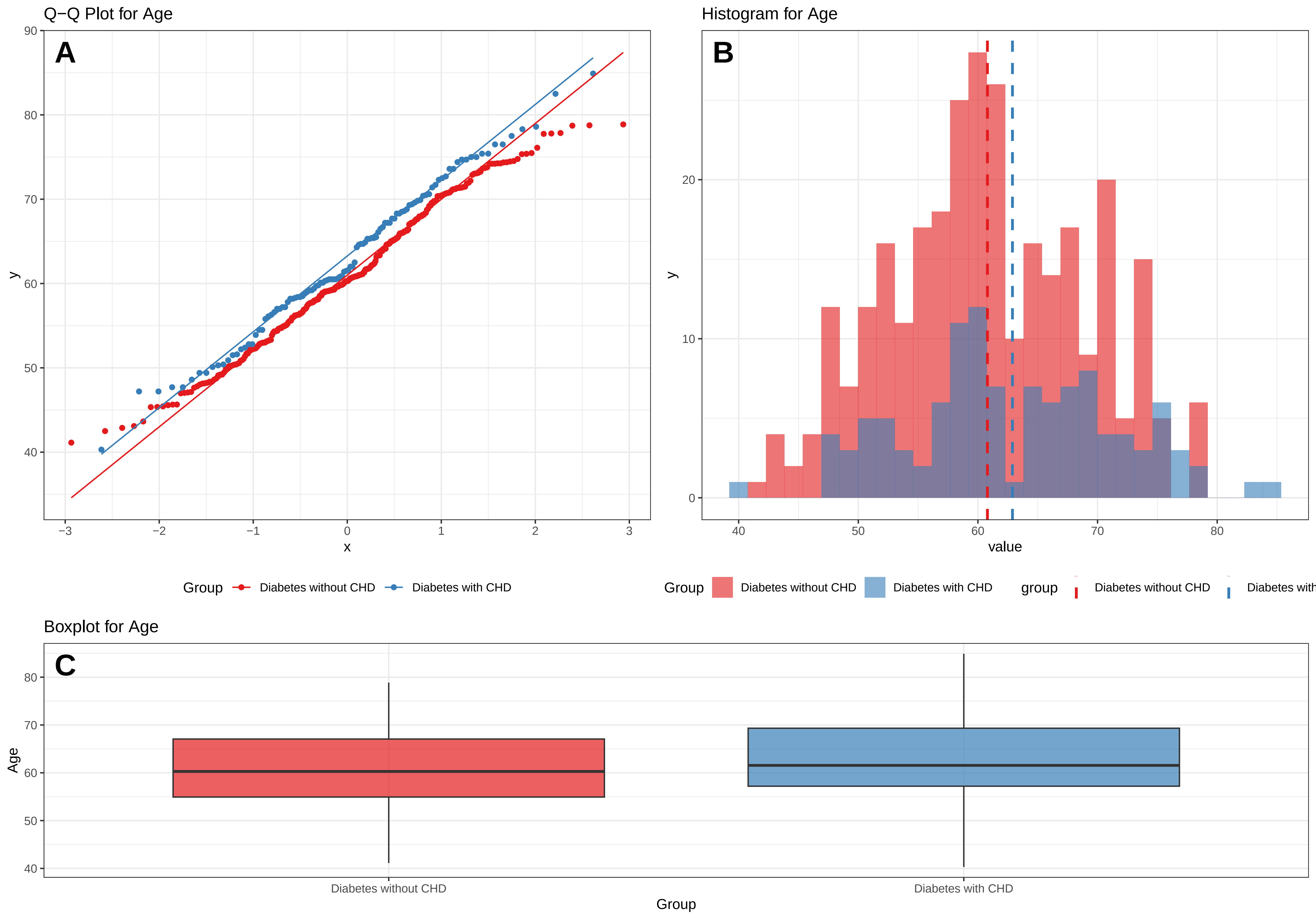

Fig. S1. Normality assessment of Age (A: Q−Q plot; B: Histogram; C: Boxplot).

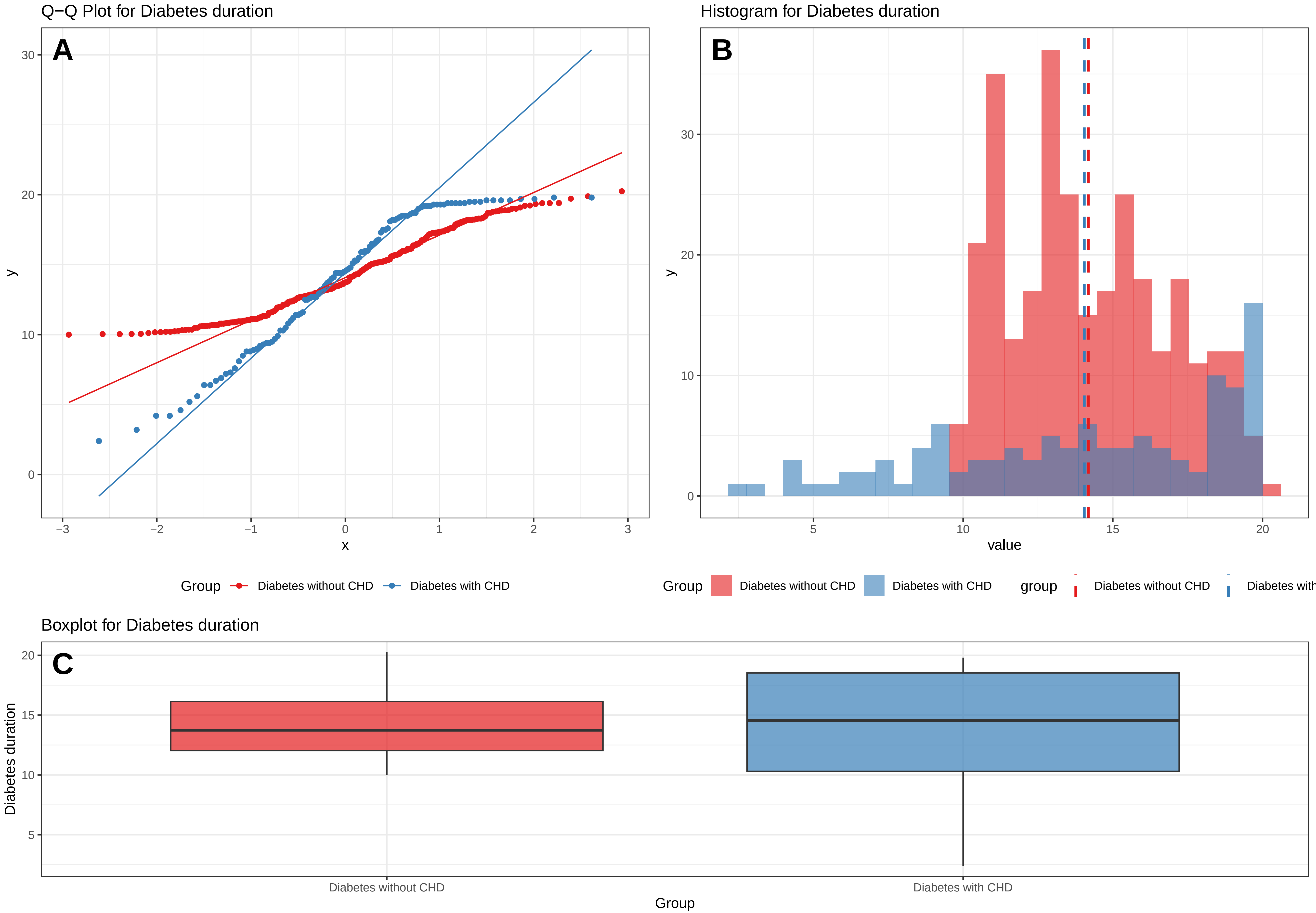

Fig. S2. Normality assessment of Diabetes duration (A: Q−Q plot; B: Histogram; C: Boxplot).

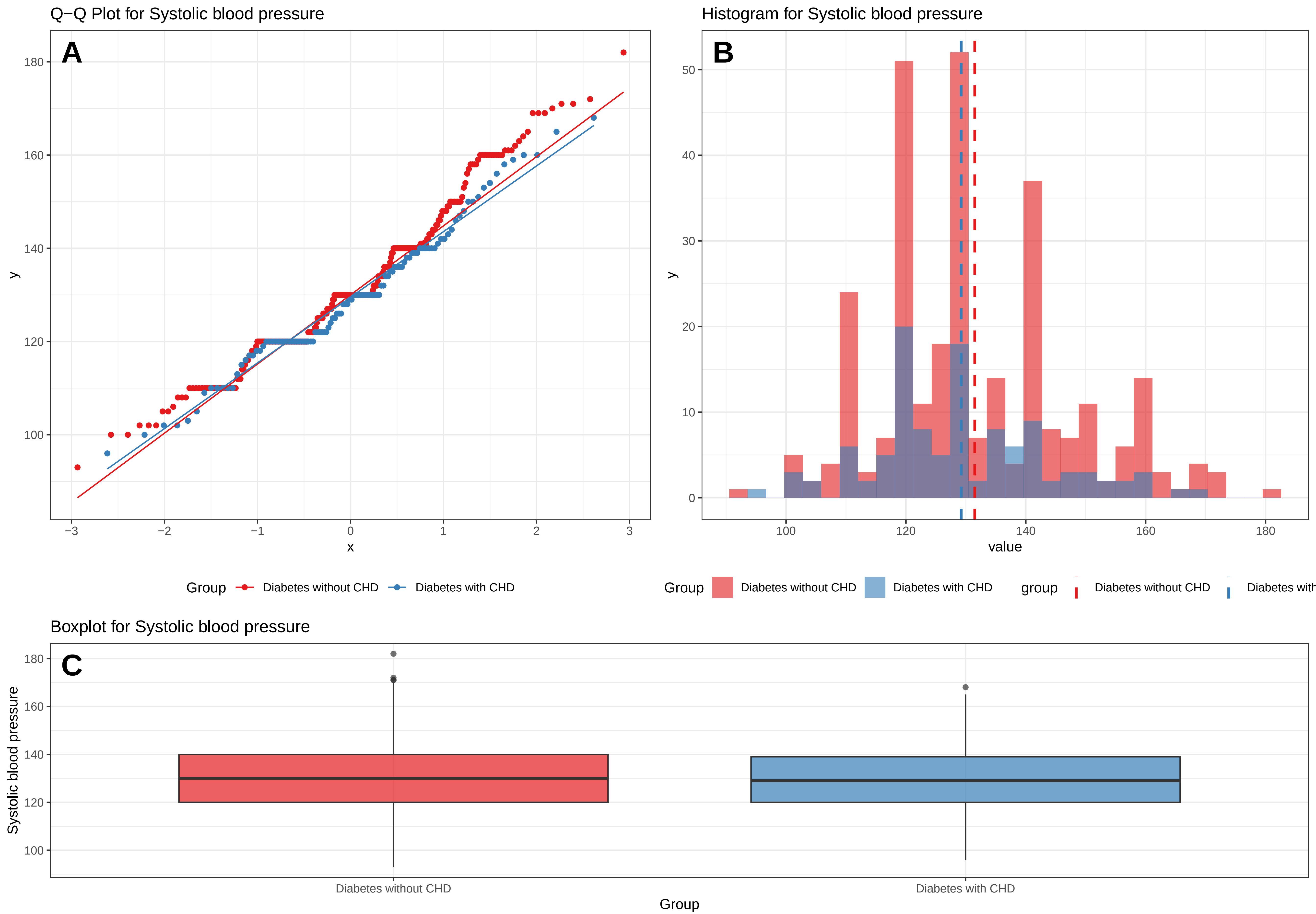

Fig. S3. Normality assessment of Systolic blood pressure (A: Q−Q plot; B: Histogram; C: Boxplot).

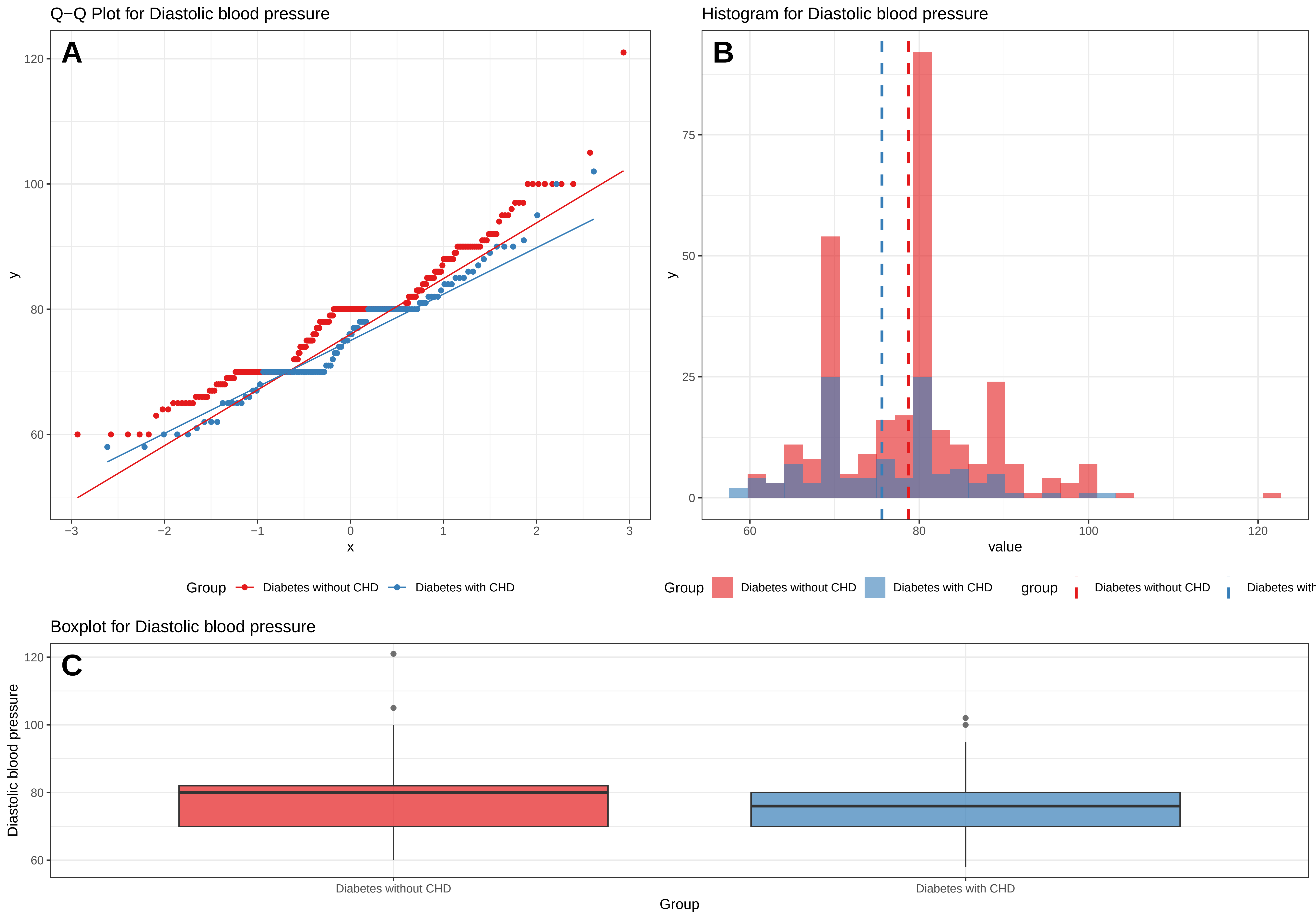

Fig. S4. Normality assessment of Diastolic blood pressure (A: Q−Q plot; B: Histogram; C: Boxplot).

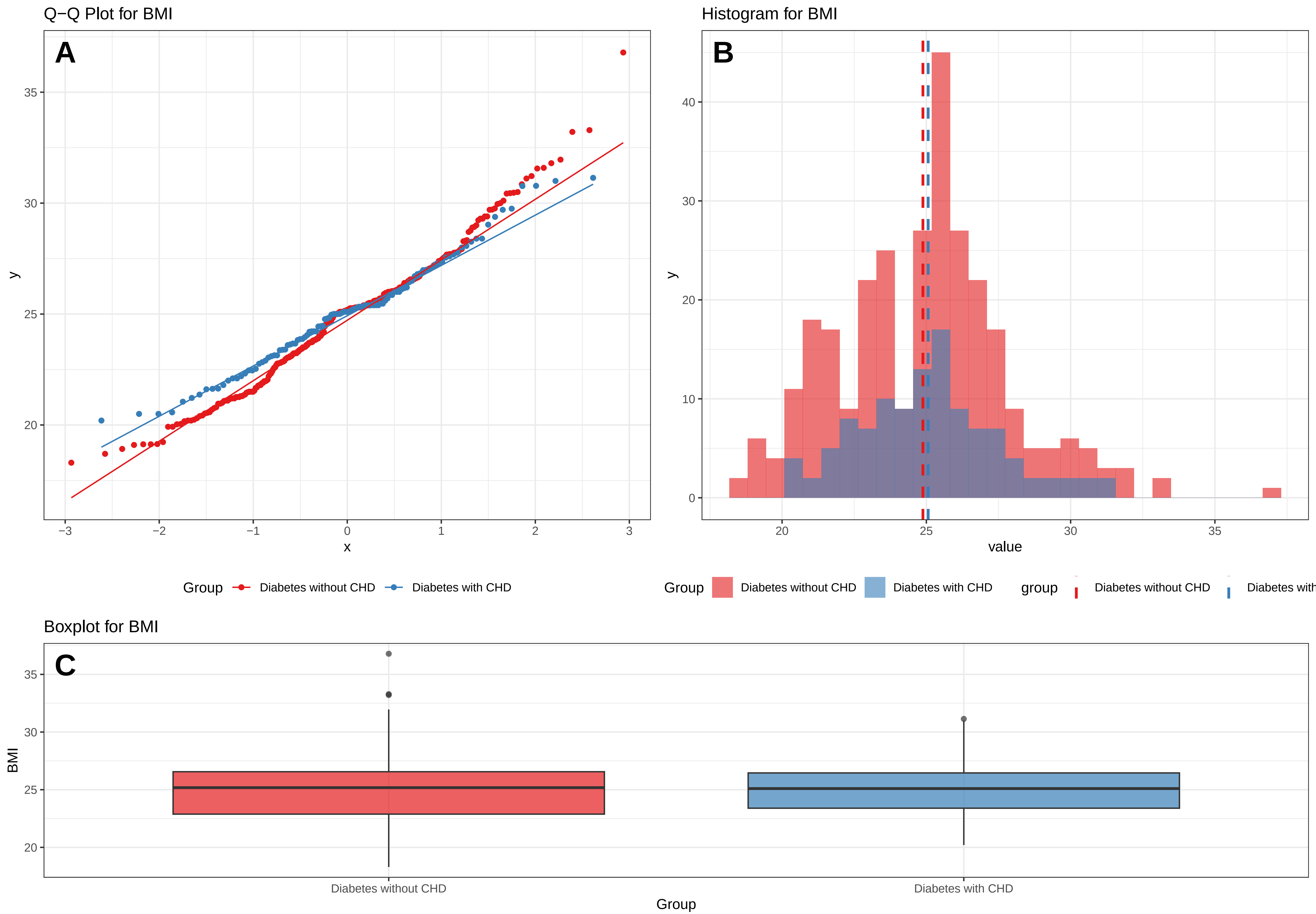

Fig. S5. Normality assessment of BMI (A: Q−Q plot; B: Histogram; C: Boxplot).

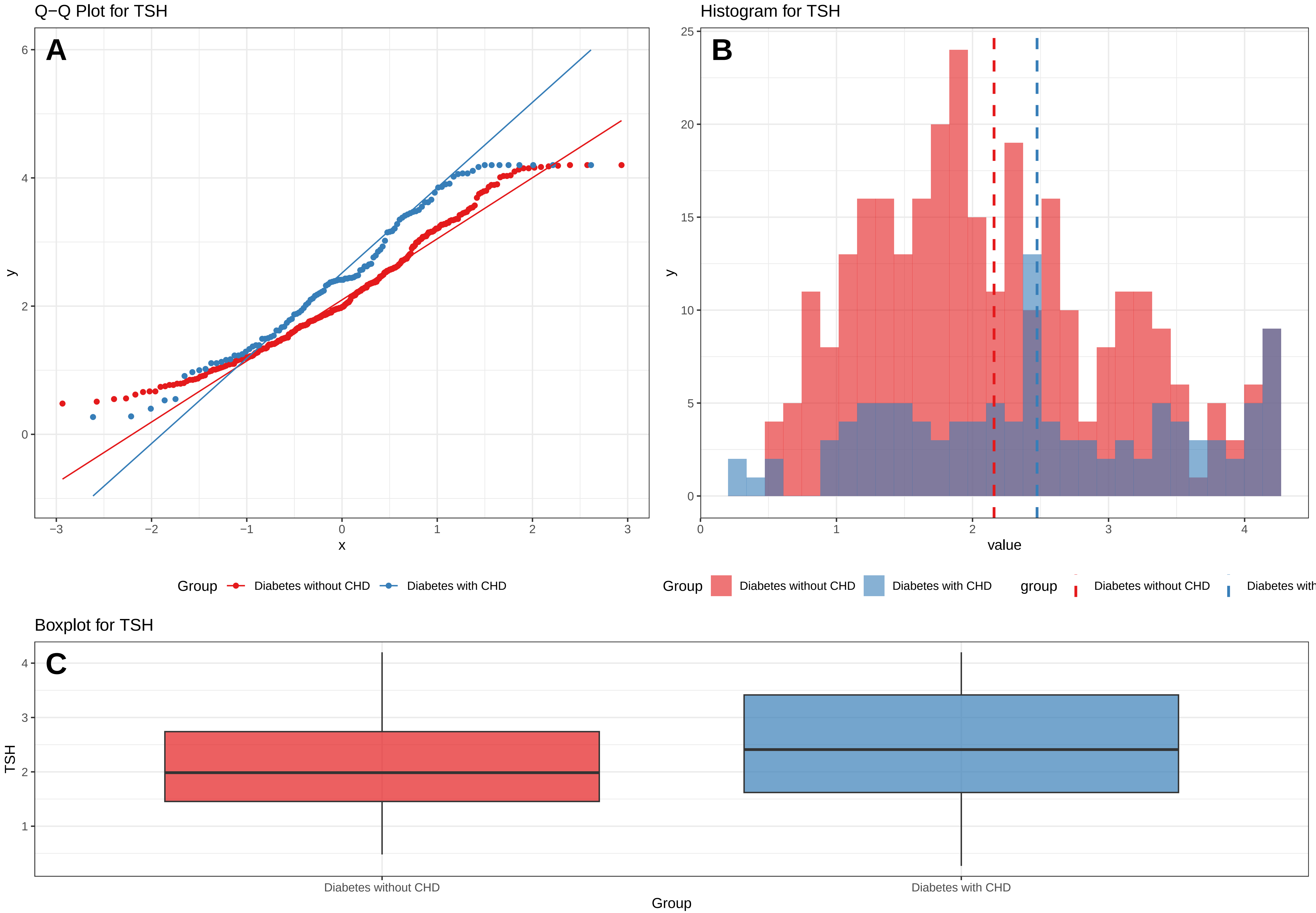

Fig. S6. Normality assessment of TSH (A: Q−Q plot; B: Histogram; C: Boxplot).

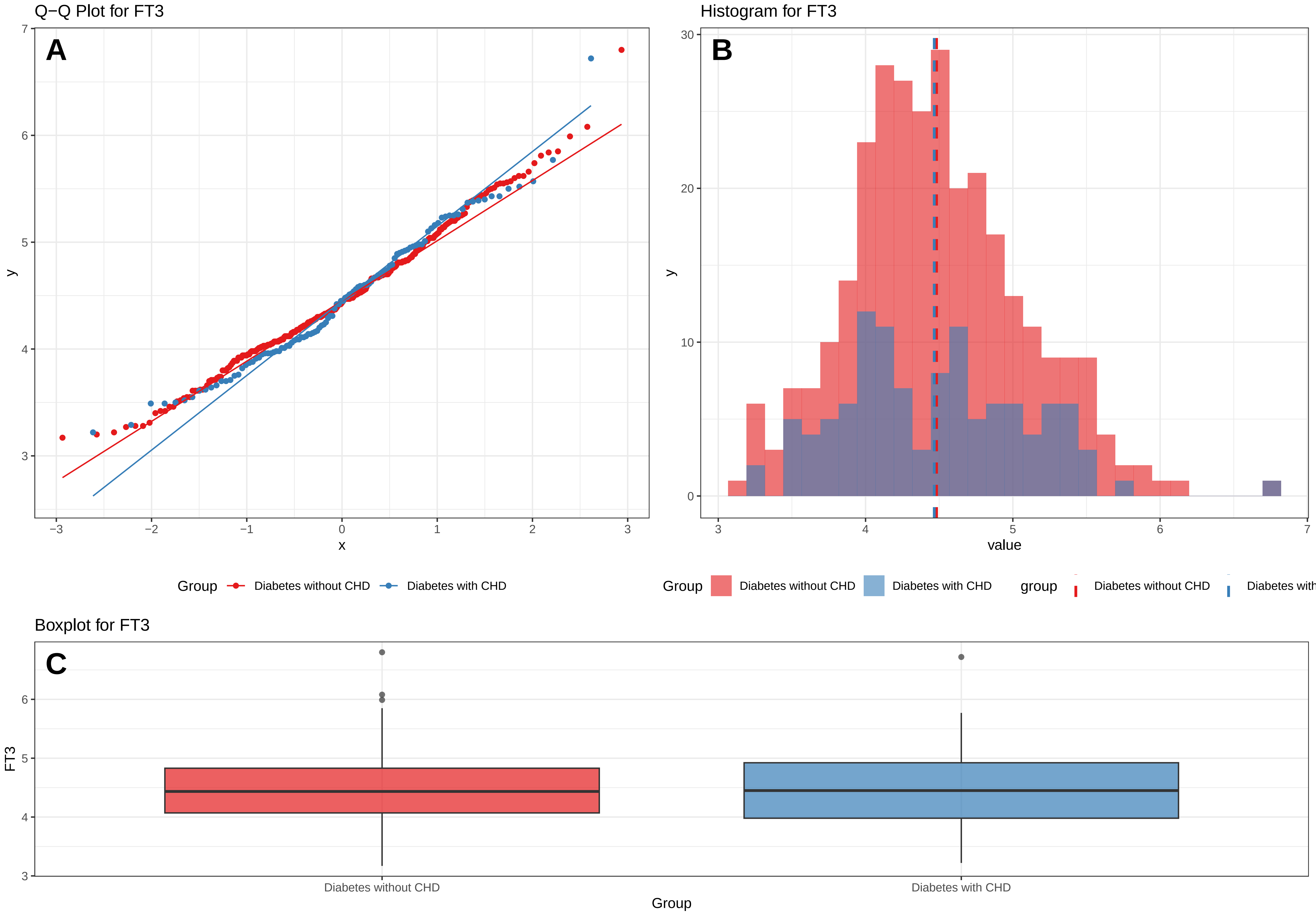

Fig. S7. Normality assessment of FT3 (A: Q−Q plot; B: Histogram; C: Boxplot).

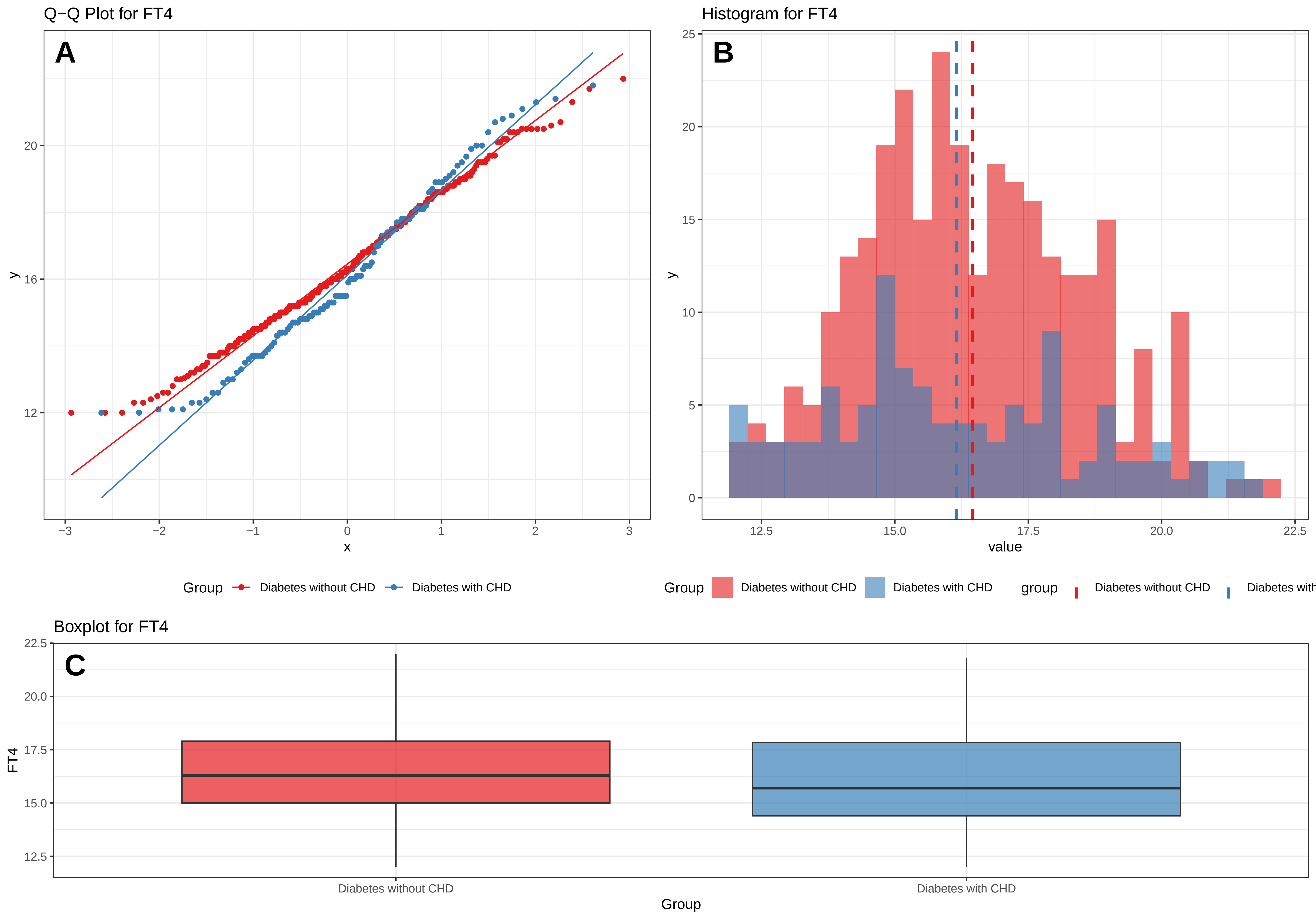

Fig. S8. Normality assessment of FT4 (A: Q−Q plot; B: Histogram; C: Boxplot).

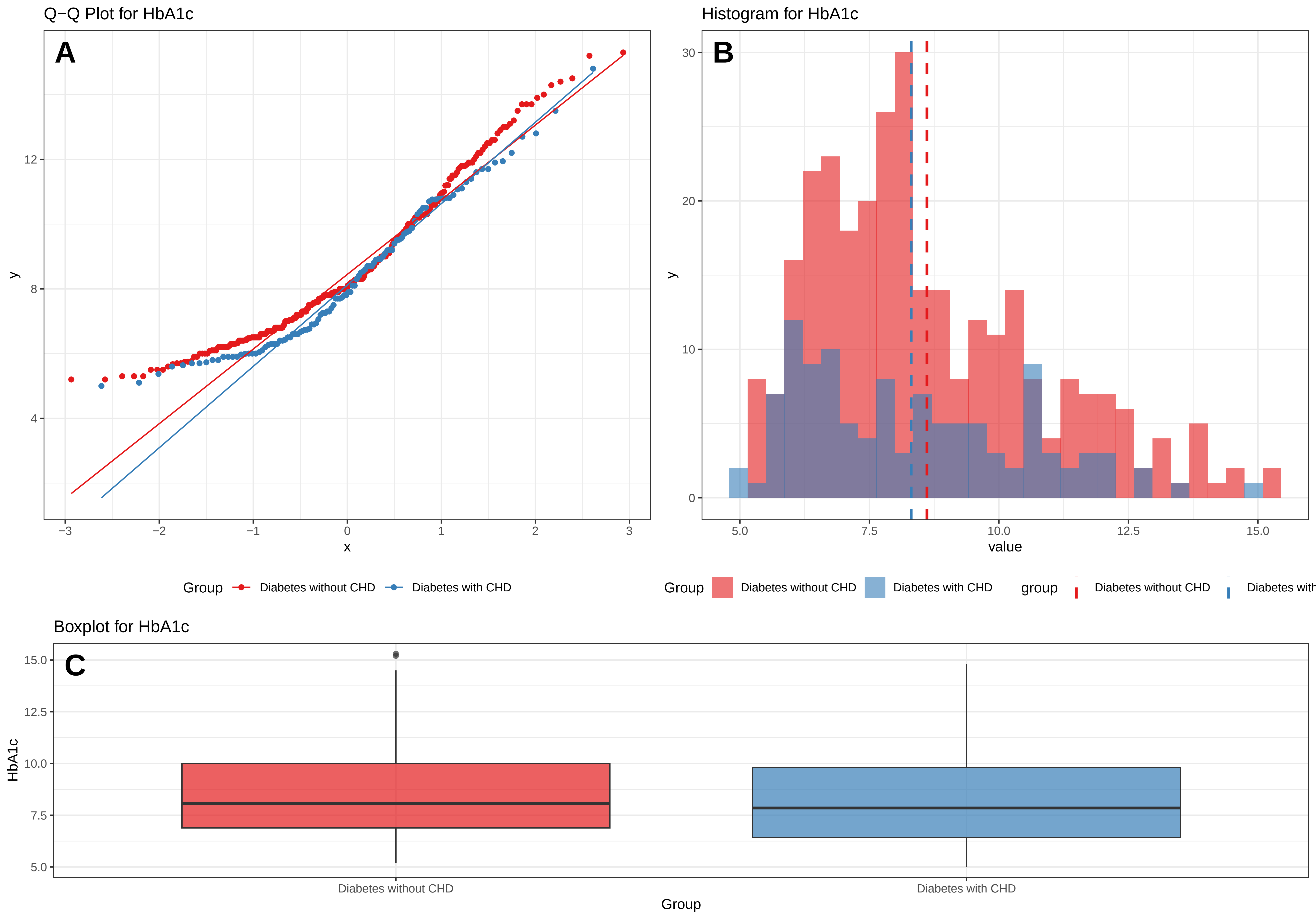

Fig. S9. Normality assessment of HbA1c (A: Q−Q plot; B: Histogram; C: Boxplot).

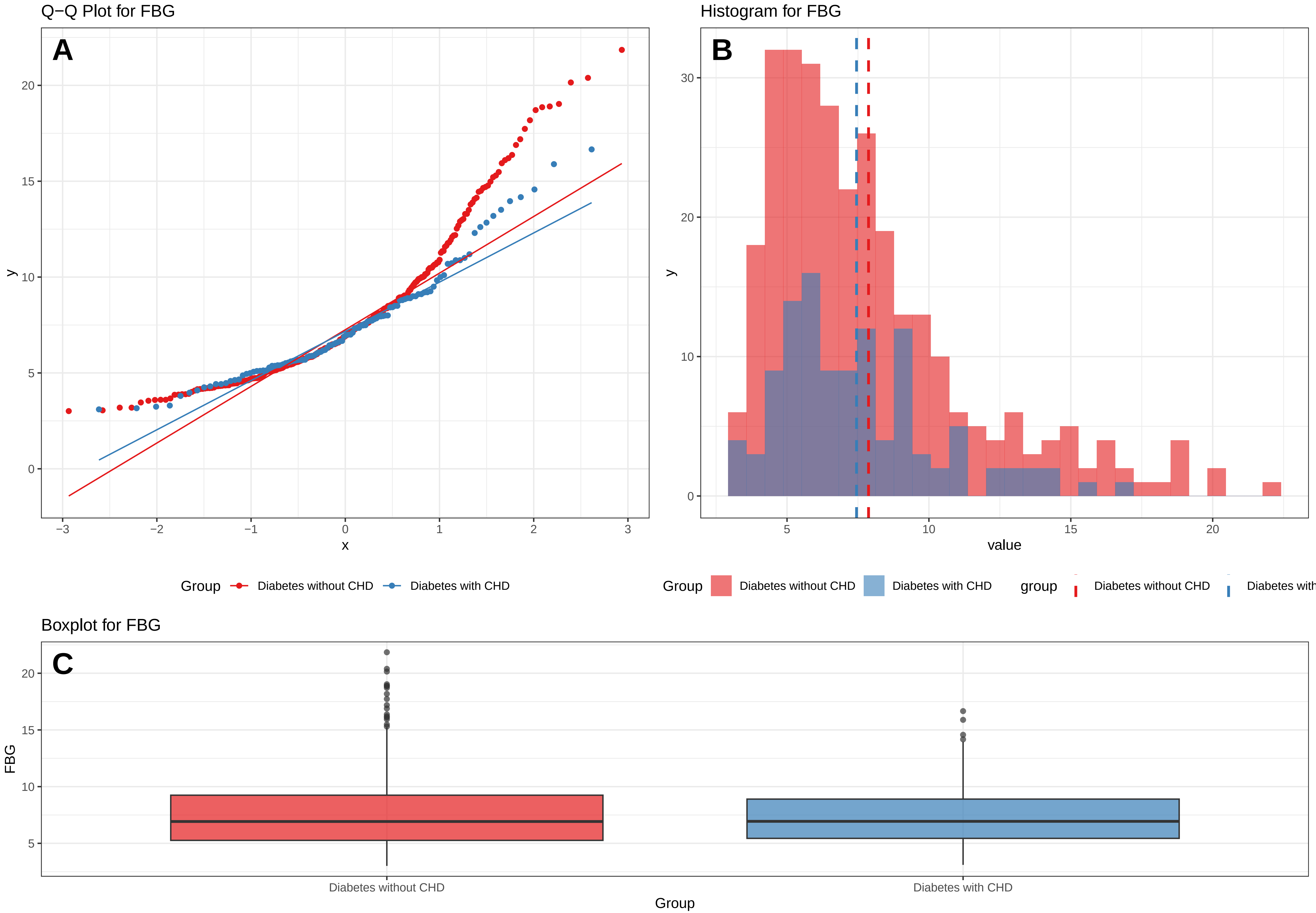

Fig. S10. Normality assessment of FBG (A: Q−Q plot; B: Histogram; C: Boxplot).

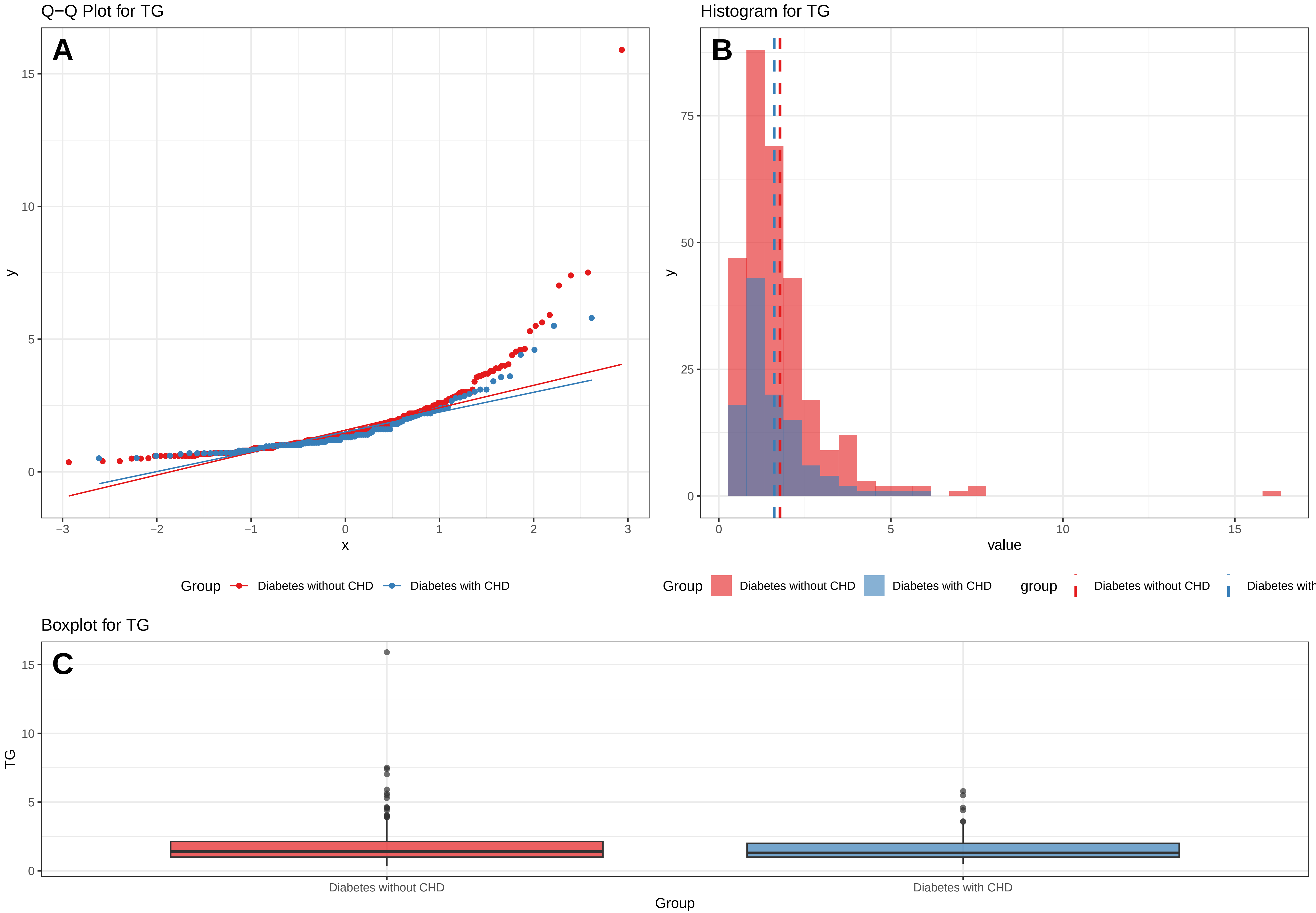

Fig. S11. Normality assessment of TG (A: Q−Q plot; B: Histogram; C: Boxplot).

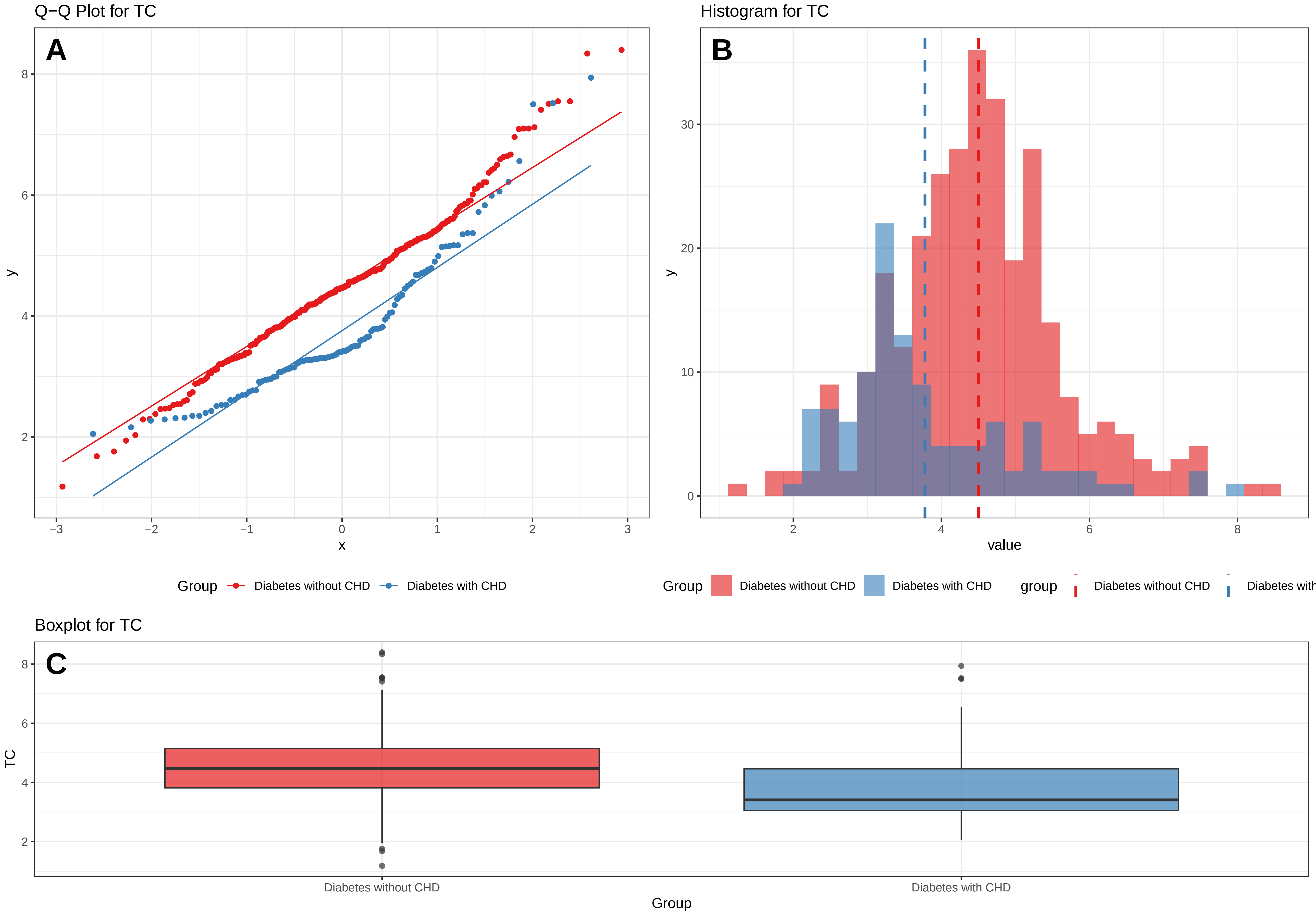

Fig. S12. Normality assessment of TC (A: Q−Q plot; B: Histogram; C: Boxplot).

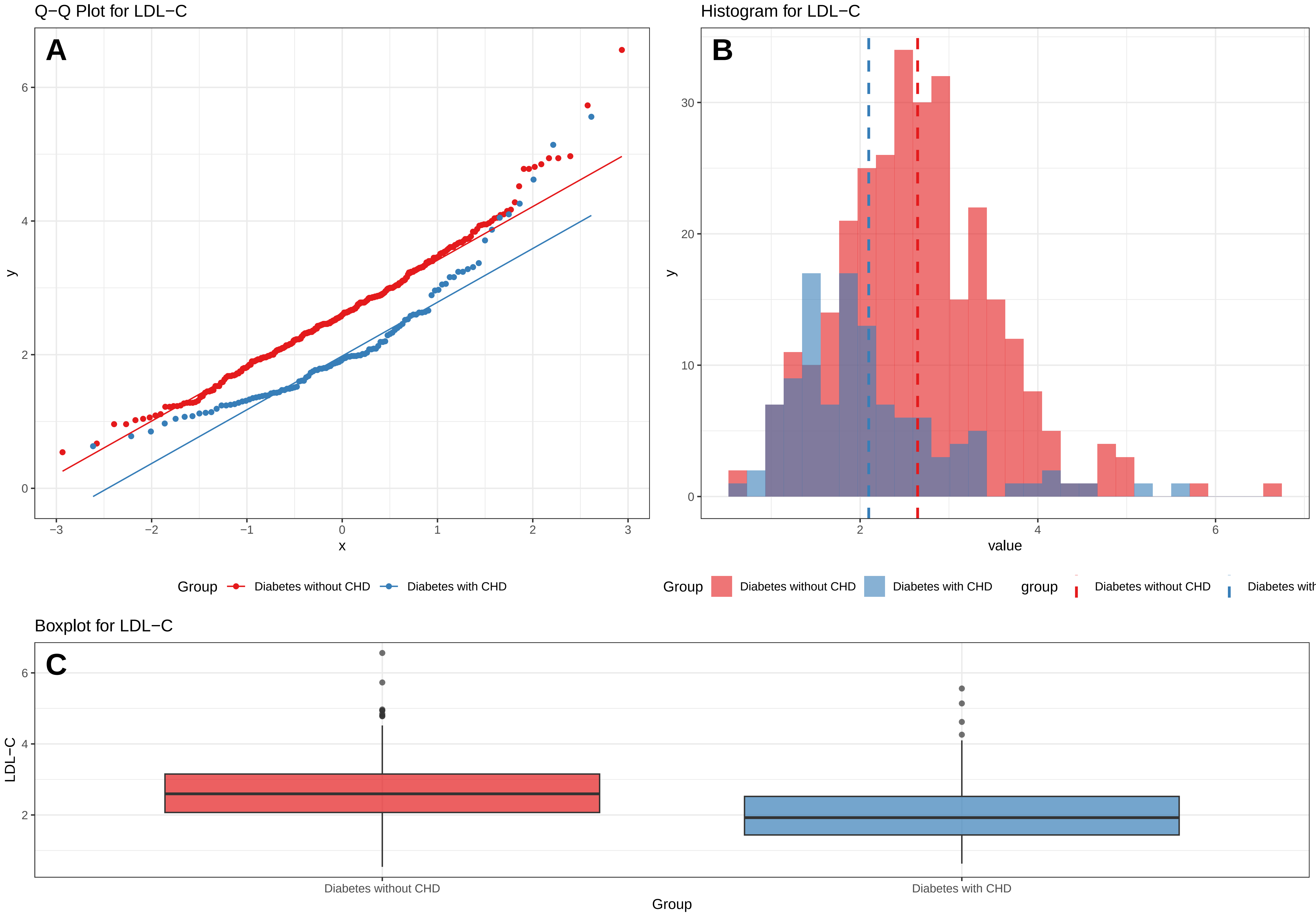

Fig. S13. Normality assessment of LDL−C (A: Q−Q plot; B: Histogram; C: Boxplot).

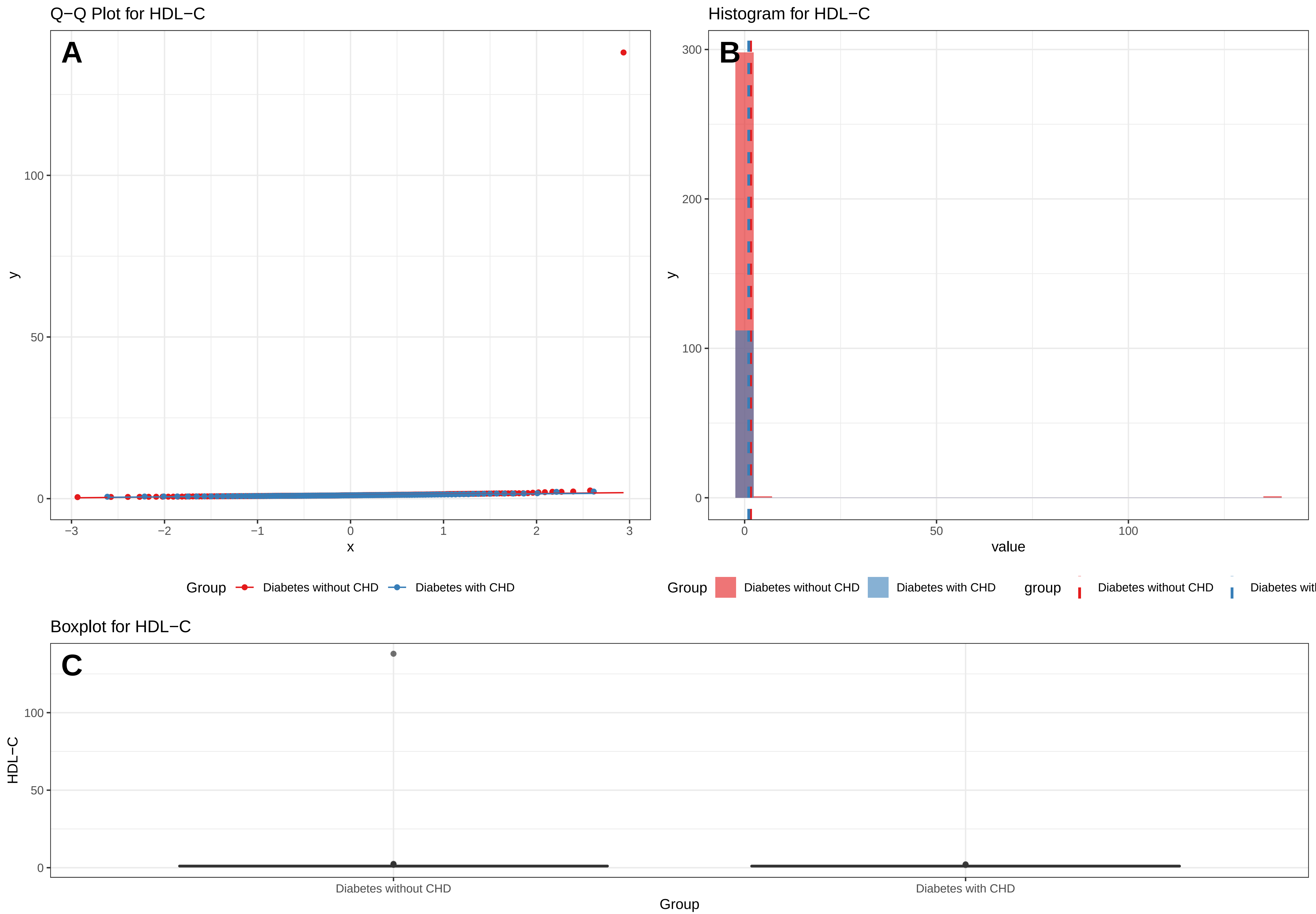

Fig. S14. Normality assessment of HDL−C (A: Q−Q plot; B: Histogram; C: Boxplot).

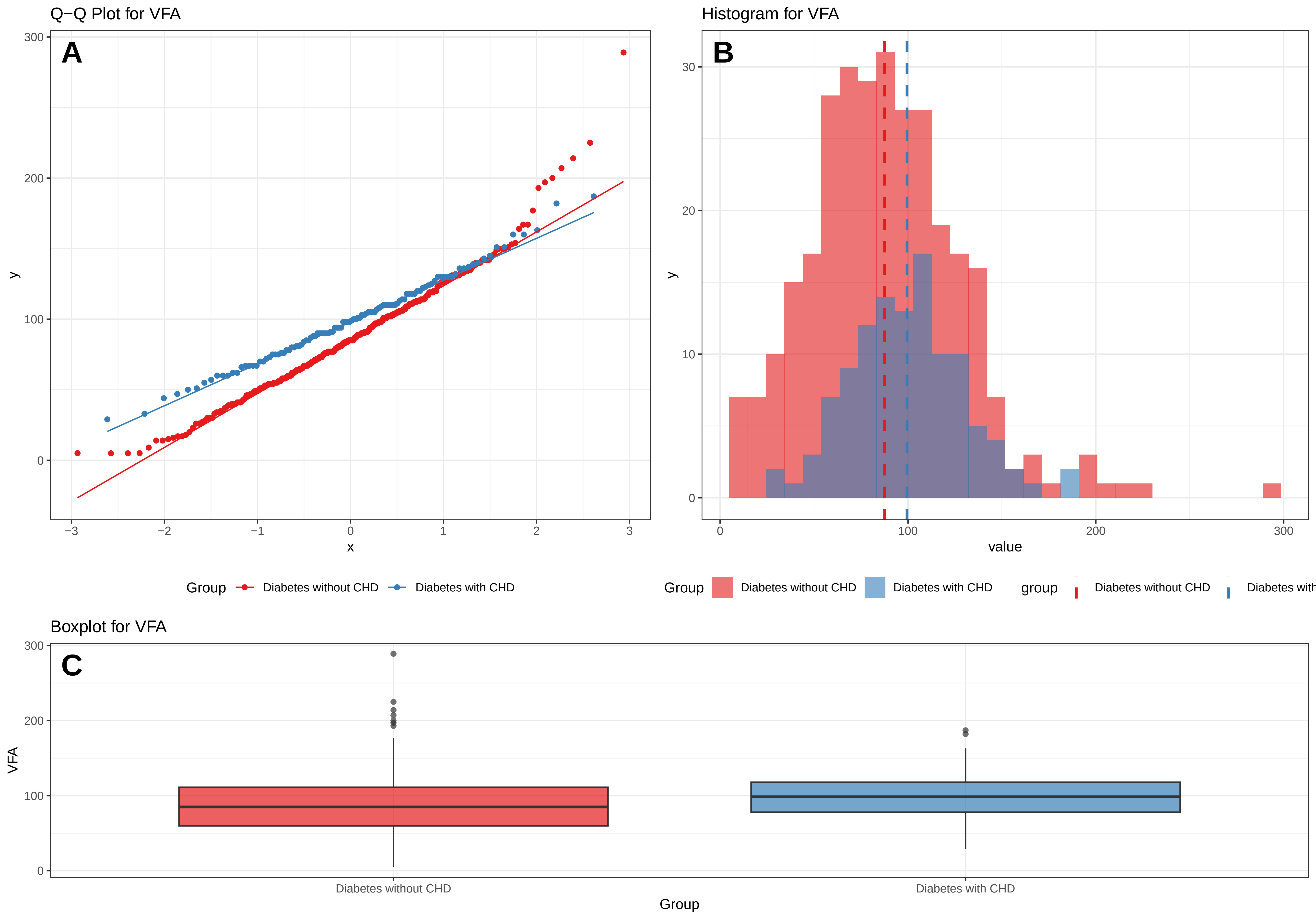

Fig. S15. Normality assessment of VFA (A: Q−Q plot; B: Histogram; C: Boxplot).

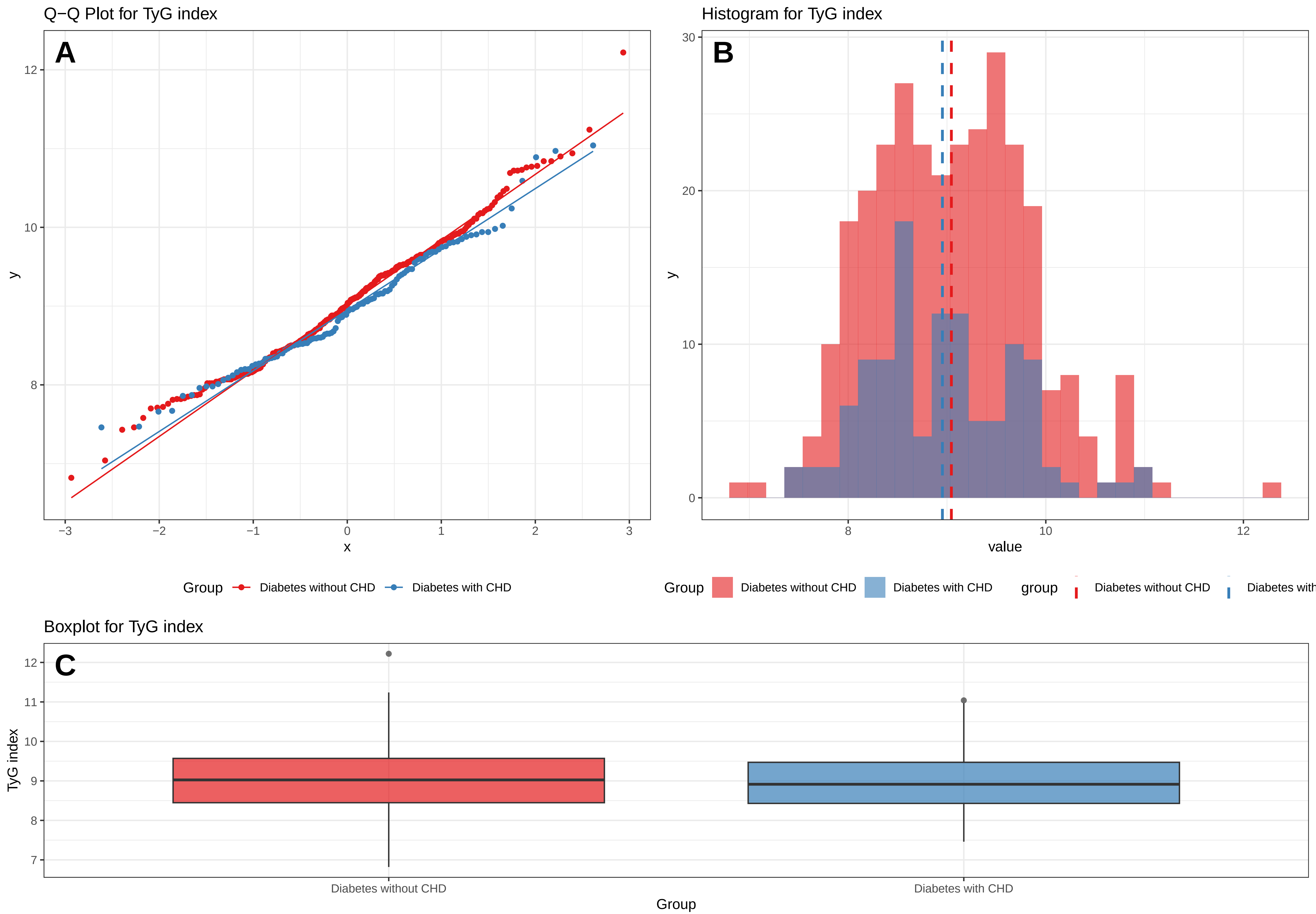

Fig. S16. Normality assessment of TyG index (A: Q−Q plot; B: Histogram; C: Boxplot).

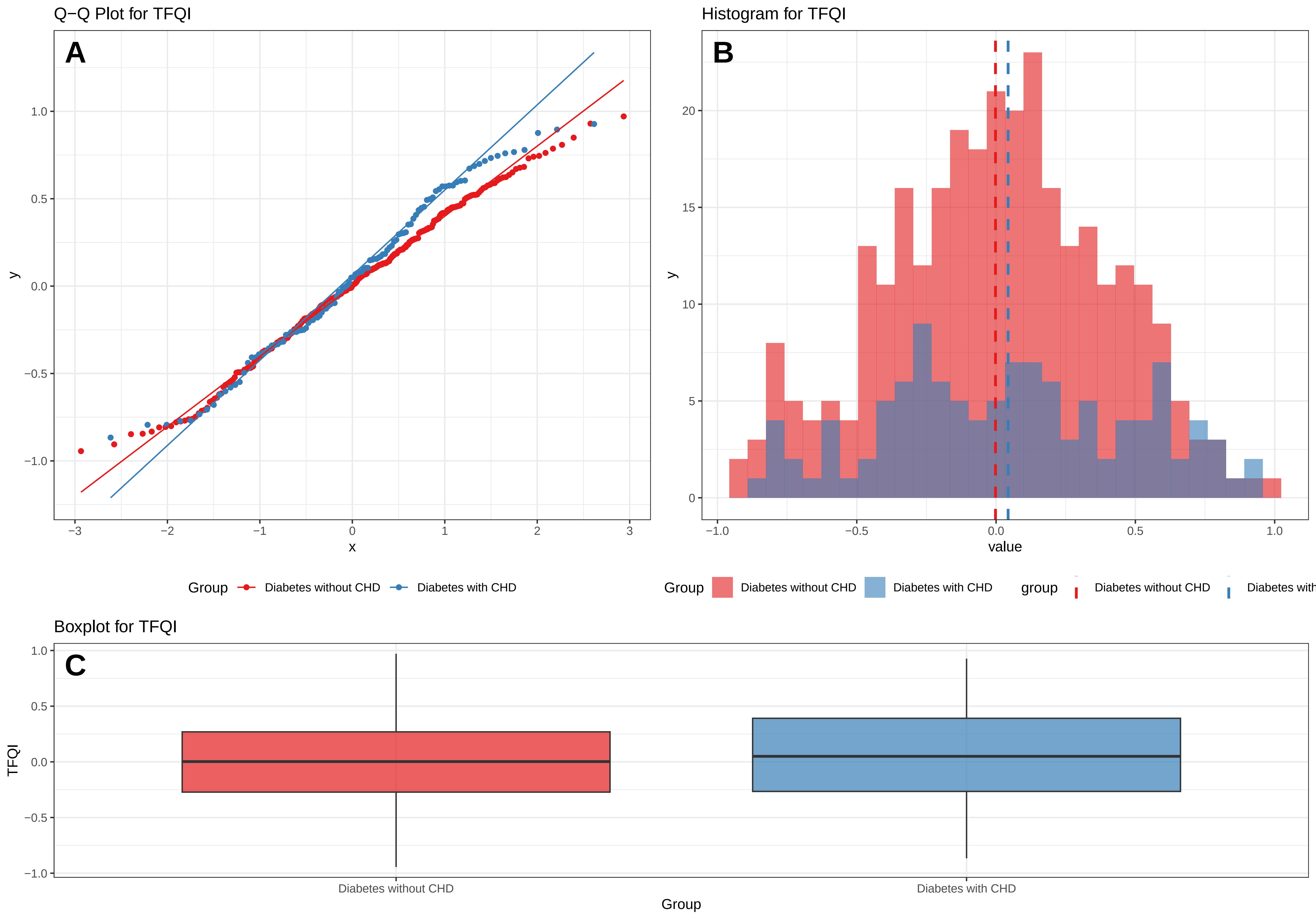

Fig. S17. Normality assessment of TFQI (A: Q−Q plot; B: Histogram; C: Boxplot).

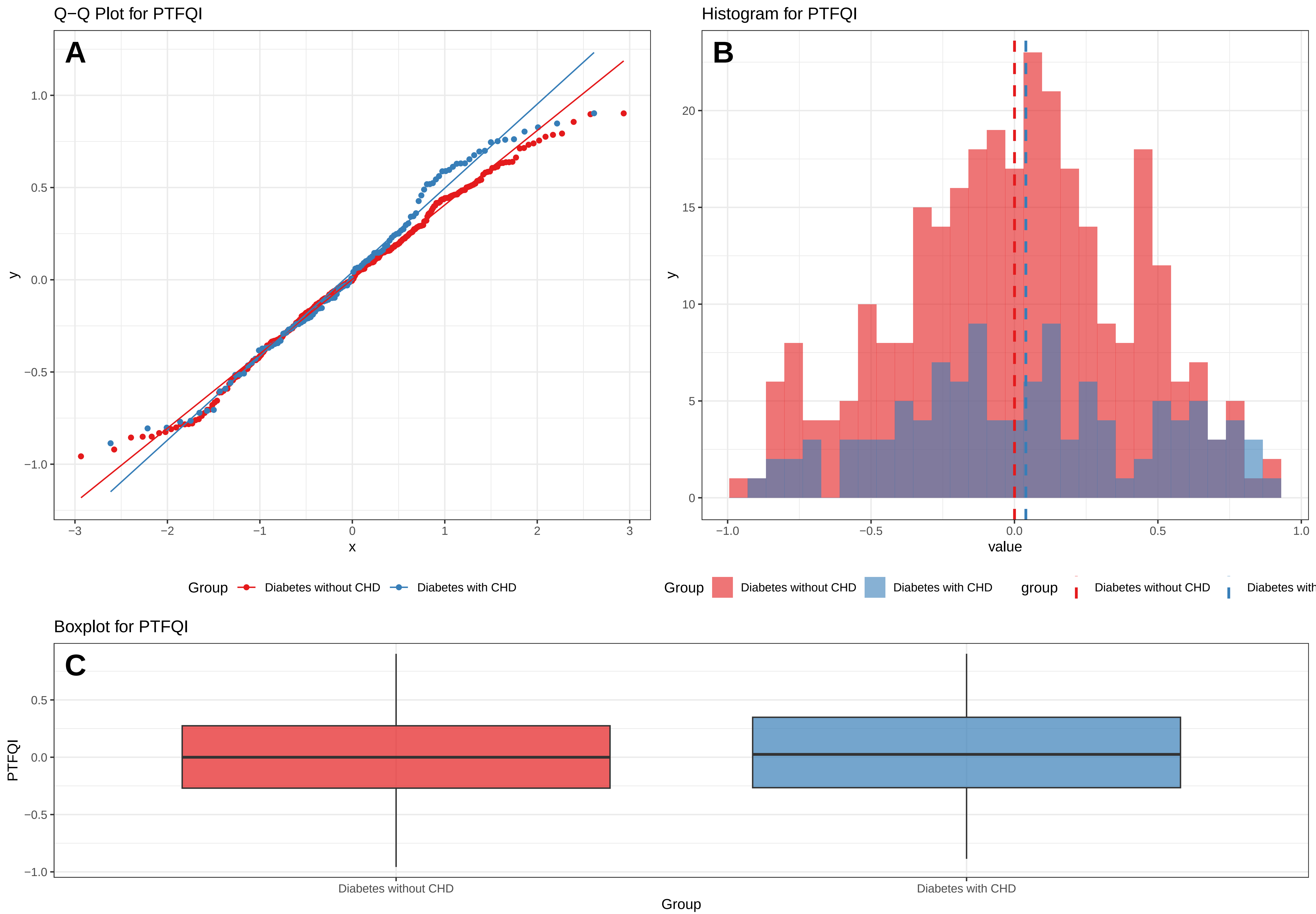

Fig. S18. Normality assessment of PTFQI (A: Q−Q plot; B: Histogram; C: Boxplot).

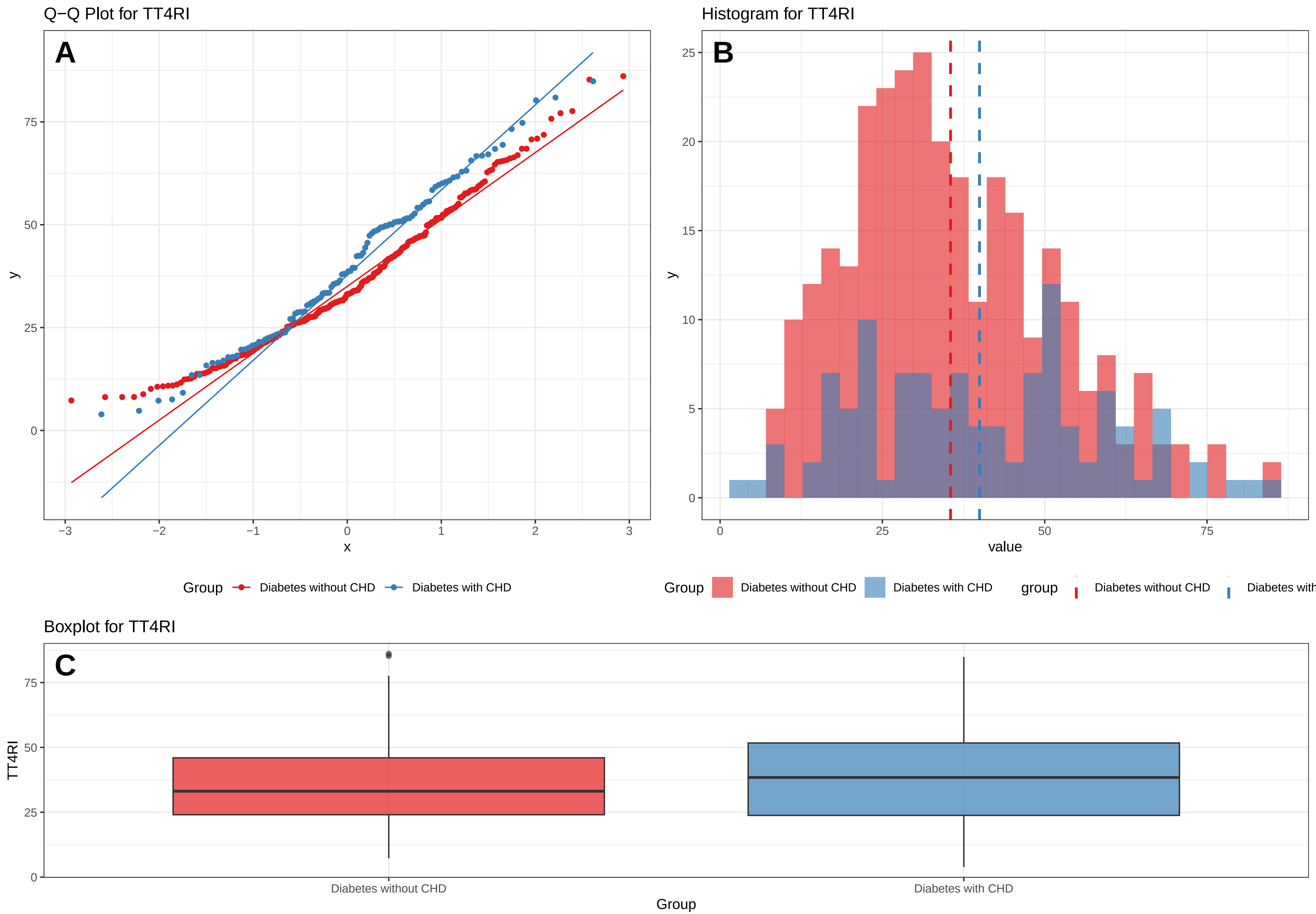

Fig. S19. Normality assessment of TT4RI (A: Q−Q plot; B: Histogram; C: Boxplot).

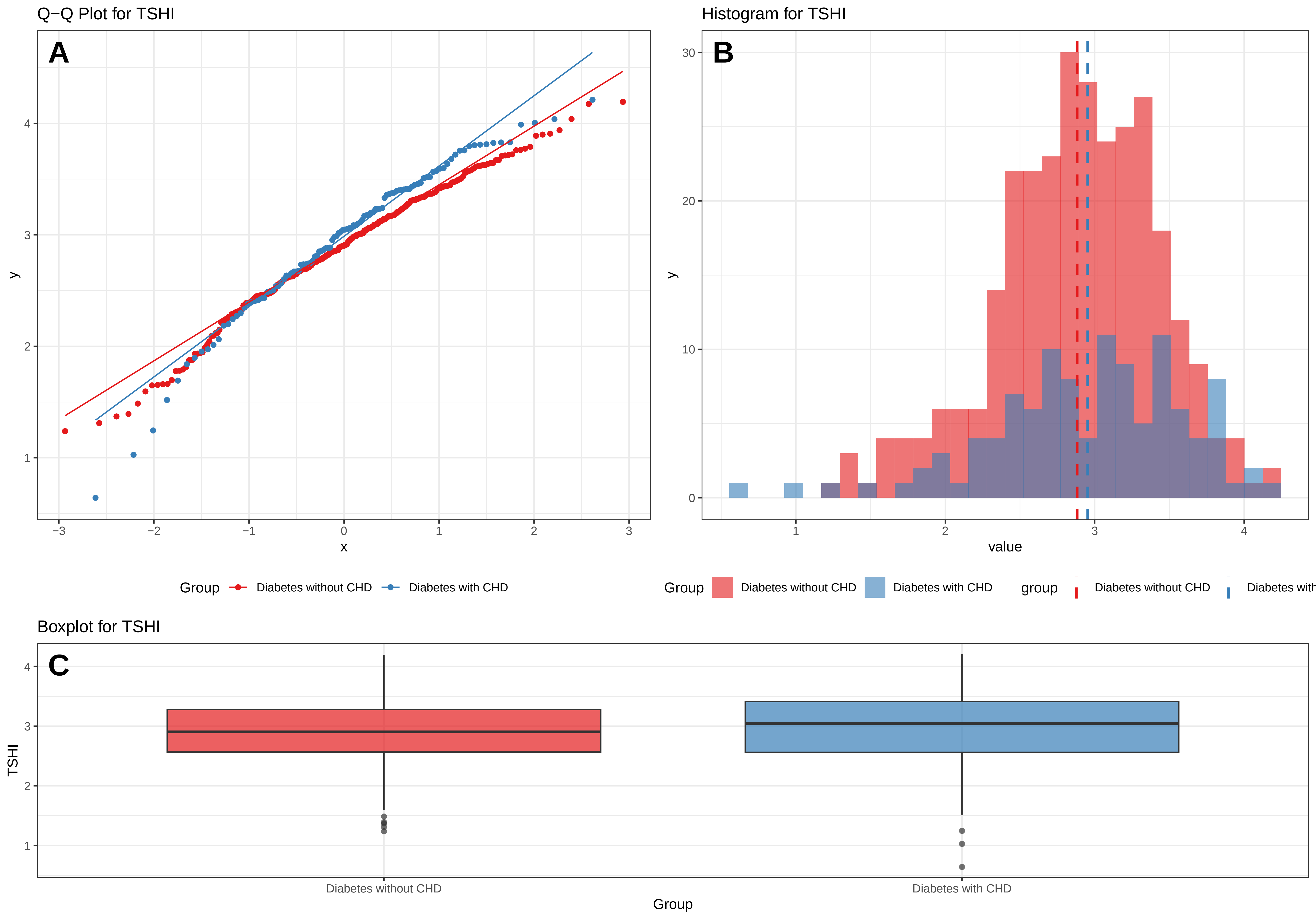

Fig. S20. Normality assessment of TSHI (A: Q−Q plot; B: Histogram; C: Boxplot).

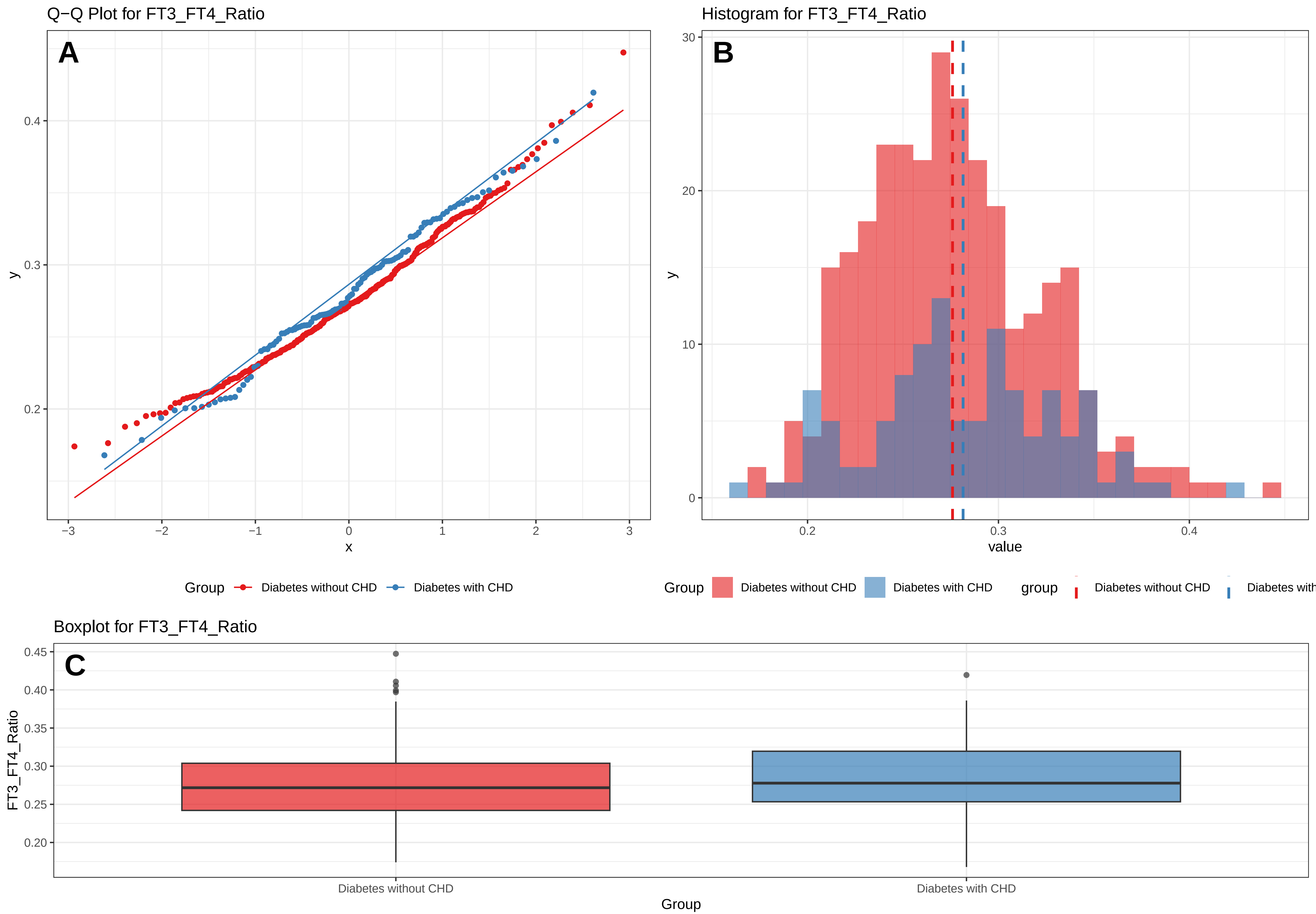

Fig. S21. Normality assessment of FT3_FT4_Ratio (A: Q−Q plot; B: Histogram; C: Boxplot).

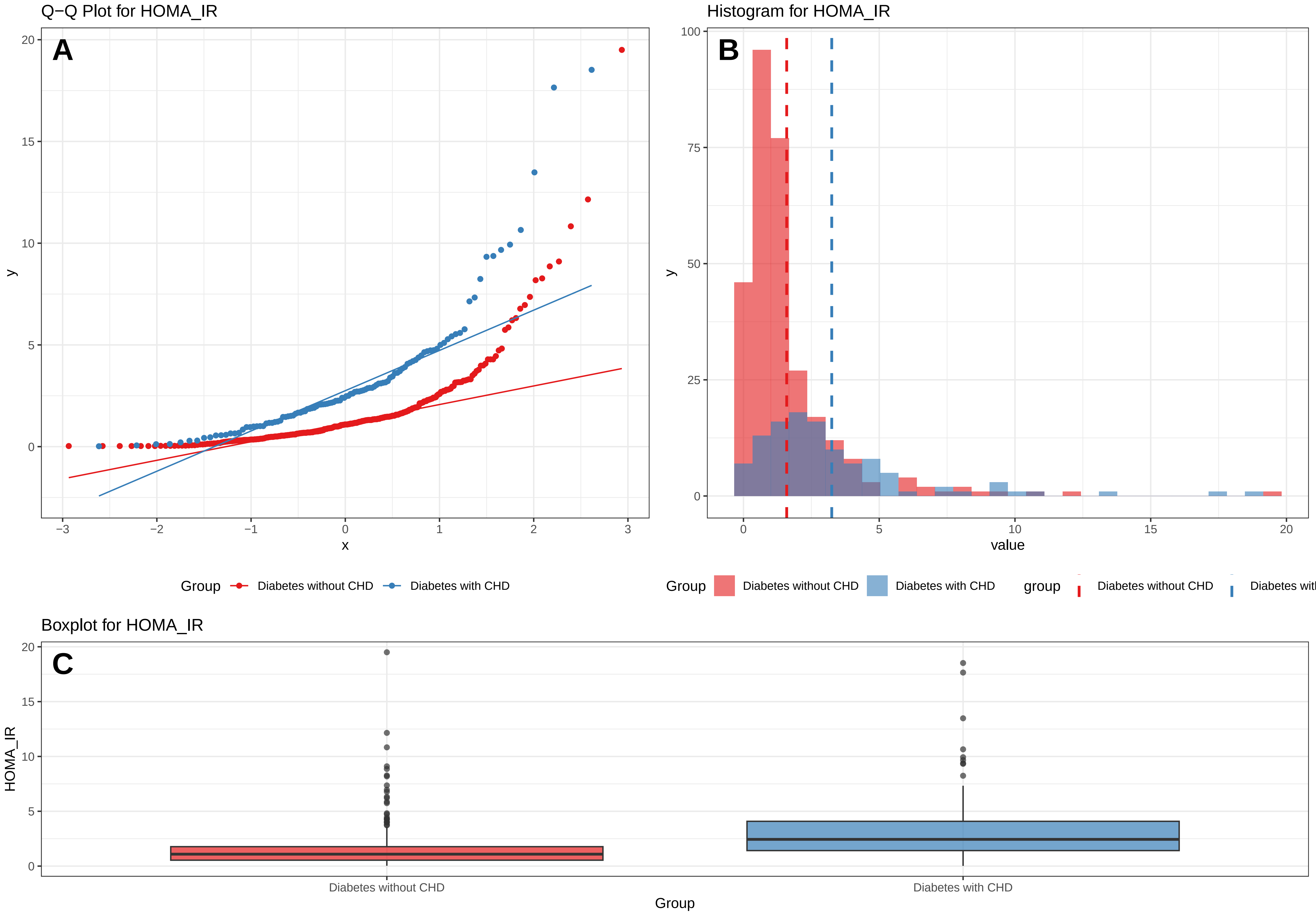

Fig. S22. Normality assessment of HOMA_IR (A: Q−Q plot; B: Histogram; C: Boxplot).

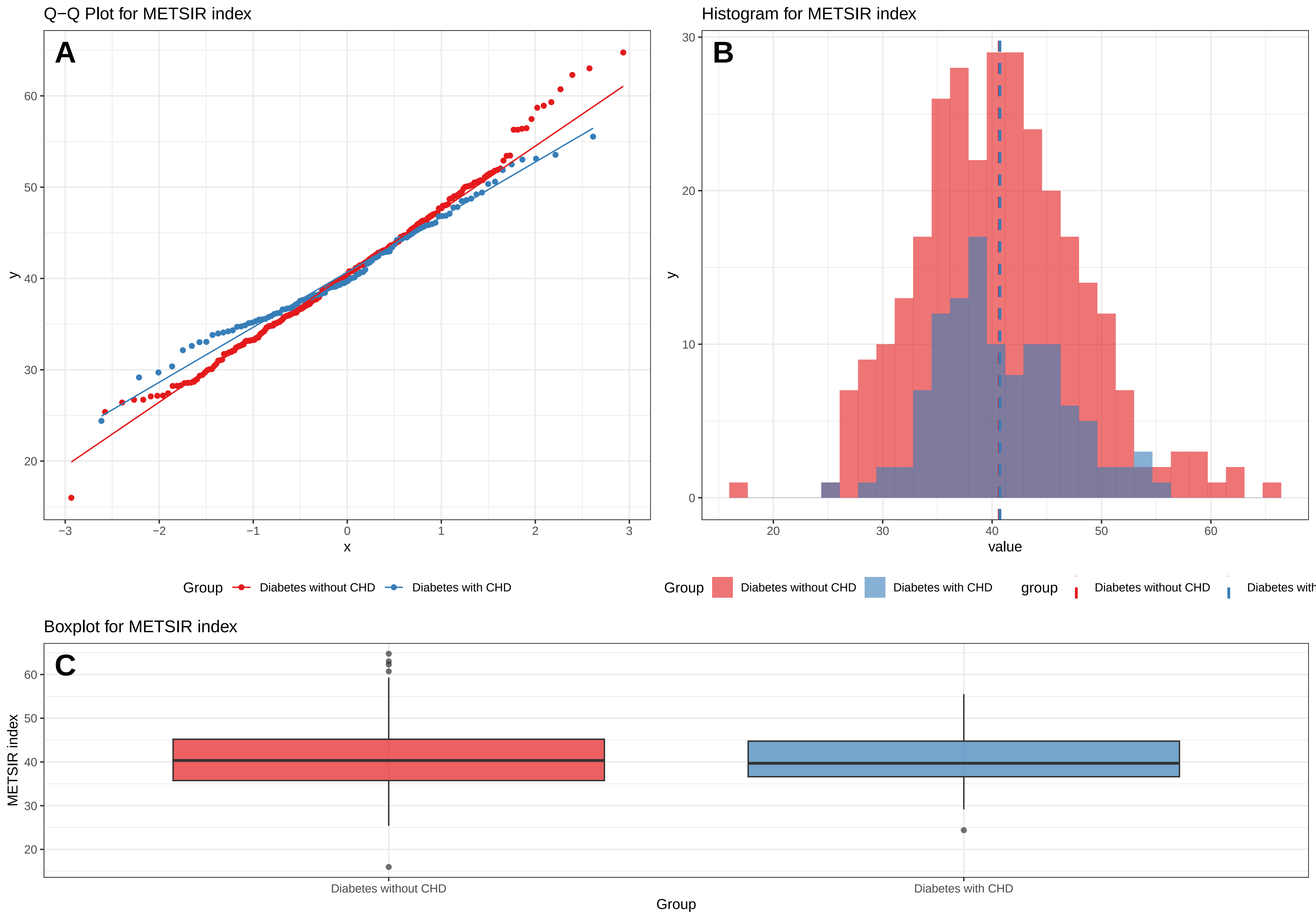

Fig. S23. Normality assessment of METSIR index (A: Q−Q plot; B: Histogram; C: Boxplot).
